# Chemical composition of material extractives influences microbial growth and dynamics on wetted wood materials

**DOI:** 10.1038/s41598-020-71560-3

**Published:** 2020-09-02

**Authors:** Dan Zhao, Cesar Cardona, Neil Gottel, Valerie J. Winton, Paul M. Thomas, Daniel A. Raba, Scott T. Kelley, Christopher Henry, Jack A. Gilbert, Brent Stephens

**Affiliations:** 1 Department of Civil, Architectural, and Environmental Engineering, Illinois Institute of Technology, Alumni Memorial Hall 228E, 3201 South Dearborn Street, Chicago, IL 60616 USA; 2 Graduate Program in Biophysical Sciences, The University of Chicago, Chicago, IL USA; 3 Department of Surgery, The University of Chicago, Chicago, IL USA; 4 Department of Pediatrics, University of California San Diego School of Medicine, San Diego, CA USA; 5 Proteomics Center of Excellence and Department of Molecular Biosciences, Northwestern University, Evanston, IL USA; 6 Department of Biology, Illinois Institute of Technology, Chicago, IL USA; 7 Department of Biology, San Diego State University, San Diego, CA USA; 8 Mathematics and Computer Science, Argonne National Laboratory, Lemont, IL USA

**Keywords:** Environmental microbiology, Environmental sciences

## Abstract

The impact of material chemical composition on microbial growth on building materials remains relatively poorly understood. We investigate the influence of the chemical composition of material extractives on microbial growth and community dynamics on 30 different wood species that were naturally inoculated, wetted, and held at high humidity for several weeks. Microbial growth was assessed by visual assessment and molecular sequencing. Unwetted material powders and microbial swab samples were analyzed using reverse phase liquid chromatography with tandem mass spectrometry. Different wood species demonstrated varying susceptibility to microbial growth after 3 weeks and visible coverage and fungal qPCR concentrations were correlated (R^2^ = 0.55). *Aspergillaceae* was most abundant across all samples; *Meruliaceae* was more prevalent on 8 materials with the highest visible microbial growth. A larger and more diverse set of compounds was detected from the wood shavings compared to the microbial swabs, indicating a complex and heterogeneous chemical composition within wood types. Several individual compounds putatively identified in wood samples showed statistically significant, near-monotonic associations with microbial growth, including C_11_H_16_O_4_, C_18_H_34_O_4_, and C_6_H_15_NO. A pilot experiment confirmed the inhibitory effects of dosing a sample of wood materials with varying concentrations of liquid C_6_H_15_NO (assuming it presented as Diethylethanolamine).

## Introduction

Buildings are complex ecosystems that contain many habitats for microbial communities^1–4^. In buildings that lack a history of water damage or exposure to excessive moisture conditions, microbial communities found on surfaces are generally considered to consist of deposited microbes originating from outdoor environments and the microbiome of human occupants, typically with minimal microbial growth^5,6^. However, most buildings experience some kind of high moisture event(s) throughout their life cycles, often resulting from rain or snow penetration, plumbing leaks, building foundation cracks, floods and extreme weather events, condensation of damp air, and/or rising dampness from the ground^7–9^. Building materials that have experienced moisture damage and/or are subjected to sustained high (i.e., > 80%) relative humidity (RH) can experience microbial growth^9^, which can generate metabolites that are toxic to humans^10,11^. Microbial growth can also cause material biodeterioration, which adversely affects their physical and mechanical properties^12^. Moreover, dampness in buildings alone is associated with a variety of adverse health outcomes^13–16^.

There are several well-known factors that influence the likelihood and extent of microbial growth on building materials, including environmental conditions, water availability, and material susceptibility to microbial growth. Decades of research have shown that microbial (especially fungal) growth on building materials is enhanced under warm and humid conditions^17–22^. Furthermore, microbial growth is also enhanced under liquid wetting (i.e., soaked) conditions compared to when high humidity is the sole moisture source^23^. Microbial growth on material surfaces is also influenced by light, available nutrients, pH value, and even by the orientation of the material^24,25^. Available surface water also plays an important role in microbial growth on materials. Common building and furnishing materials such as plywood, oriented strand board (OSB) sheathing, and gypsum board are hygroscopic and will absorb trapped moisture, making them highly susceptible to fungal growth^26^. On the other hand, many other materials are hydrophobic and are far less susceptible to fungal growth, such as glass, ceramic products, polymer-based materials such as polystyrene, and others^21,27^.

Past research has shown that material composition appears to be a key driver of microbial growth susceptibility. For example, materials such as ceiling tiles, wood, and gypsum board paper backing, which are organic or are produced from organic products, have been shown to provide ample nutrients to support fungal growth when held at high moisture conditions, while paper-free materials such as inorganic ceiling tiles and gypsum itself support little or no growth^28^. Material composition, including the presence of organic matter via settled dust, has also been shown to influence fungal abundance and enzyme activity of fungal species^29^, as well as the composition and structure of fungal communities and the relative abundance of specific genera on materials^30^. Specific chemical components that are widely considered to encourage fungal growth on materials include natural organic polymers such as lignin, cellulose, hemicellulose, pectin, and starch, which fungi can break down and utilize as a nutrient source^31,32^. Additionally, wood extractives are non-structural wood molecules that represent a minor fraction in wood and that can be removed from wood by solvents. However, they are a key source of diverse molecules, including those that are putatively bioactive^33^. The composition of extractives in wood varies widely from species to species and can vary depending on geographical origin and from which part of the tree a sample originates^34–36^.

Of particular interest to this study, many wood-based materials have been shown to have high microbial growth susceptibility, albeit with high variability between different wood species^37^. For instance, pine plywood and paper-covered gypsum board have been identified to have high fungal growth susceptibility. The large amount of sapwood in pine plywood, which has a relatively high free sugar content, and the starch adhesive used to glue the paper layers of paper-covered gypsum board, likely contribute significantly to their susceptibility to microbial colonization and growth^26^. Conversely, several wood-based materials have been shown to have decay-resistant properties. For example, yellow-cedar heartwood contains compounds that inhibit decay^38^ and Norway spruce heartwood was found to be relatively resistant to microbial growth as well^39^. One recent study assessed the relationship between fungal growth susceptibility of wood-plastic composites and volatile chemical components of the samples, finding that several compounds identified in a head space analysis above the materials were associated with higher fungal growth resistance in some wood samples (e.g., 8-propoxy-cedrane, cedrol, α-cedrene and β-cedrene in *C. lanceolate*, or China fir), while other compounds were associated with lower fungal growth resistance in other wood samples (e.g., longifolene, caryophyllene and α-pinene in *P. massoniana*, or Masson’s pine)^40^.

Previous studies have demonstrated that the decay-resistance of wood could be determined by the extractive content and its chemical compounds. For example, a strong correlation has been established between wood durability and extractive content and diversity^41,42^. For instance, an important extractive compound in teak wood, napthoquinone, was found more consistently correlated with higher decay resistance, implying that napthoquinone imparted decay resistance to teak wood against two brown-rot fungi *Polypomus palustris* and *Gloeophyllum trabeum*^43^. Also, the methonal extracts of Alaska cedar wood and western juniper wood showed significant antimicrobial activity against test microbes, including *Fusobacterium necrophorum, Clostridium perfringens, Actinomyces bovis* and *Candida albicans*^44^.

Despite these findings, there still remains a lack of understanding of the fundamental chemical drivers of microbial growth susceptibility in wood extractives and how variability in wood extractives chemical composition influences microbial growth and community composition when subject to high moisture conditions. Therefore, the objective of this study is to investigate the influence of the chemical composition of wood extractives on microbial growth and dynamics on a diverse set of wetted wood material samples using small-scale chamber experiments.

## Results

### Visible microbial growth and fungal qPCR

**Figure 1:**
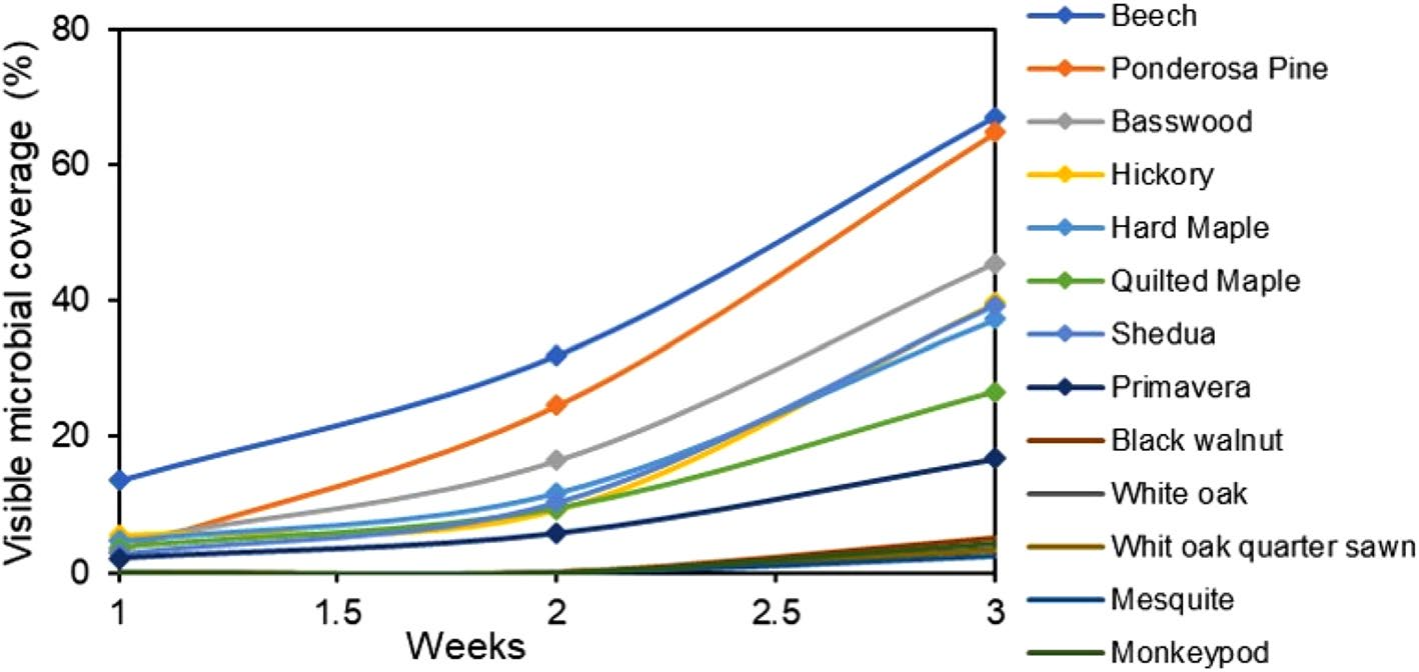
Fractional area of microbial growth coverage over time on 13 wood species with visible microbial growth after wetting and held at 94% RH for 3 weeks. There was no visible microbial growth on the other 17 wood species in the 3-week test period.

**Figure 2:**
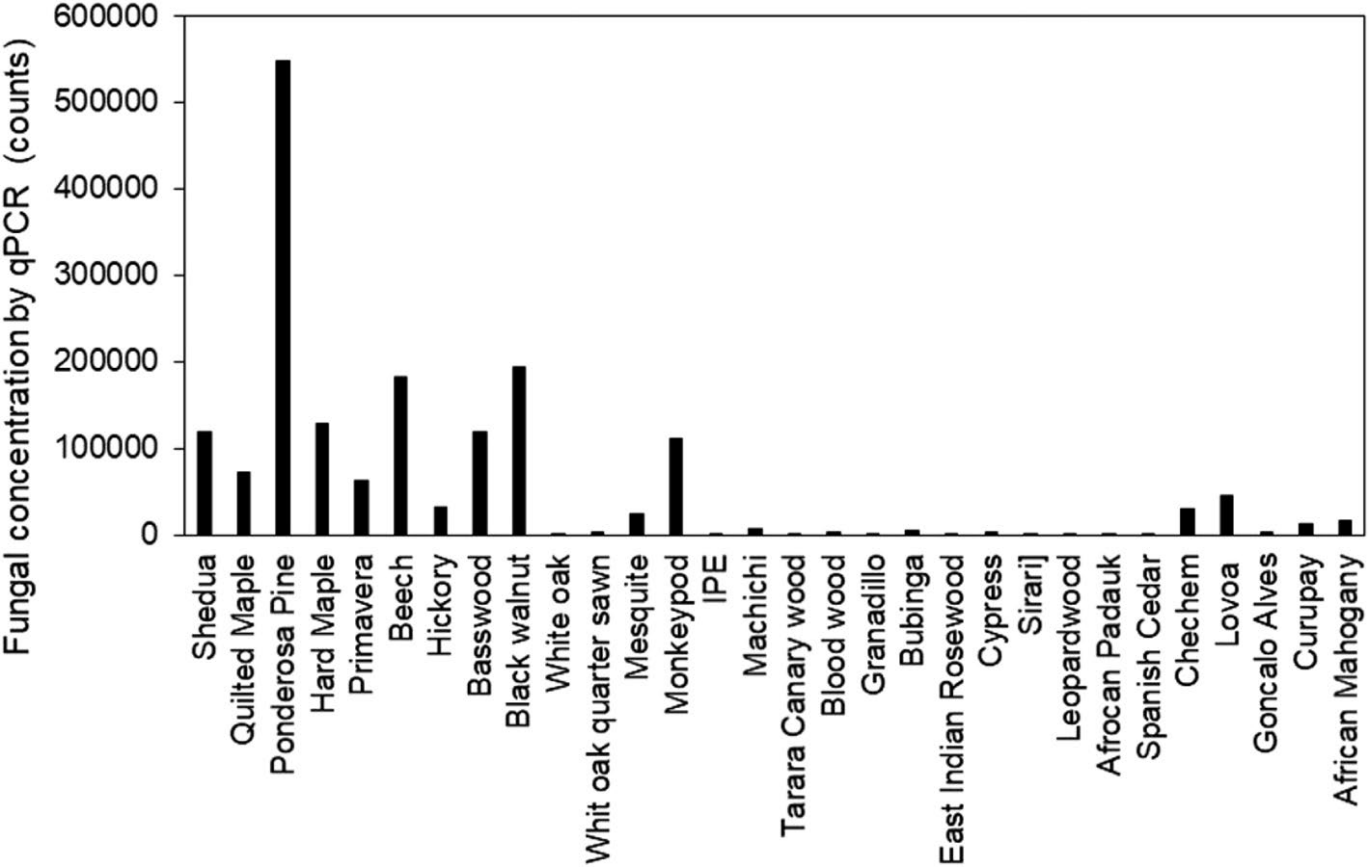
Fungal qPCR concentrations on all 30 wood species after 3 weeks of incubation at high RH.

**Figure 3:**
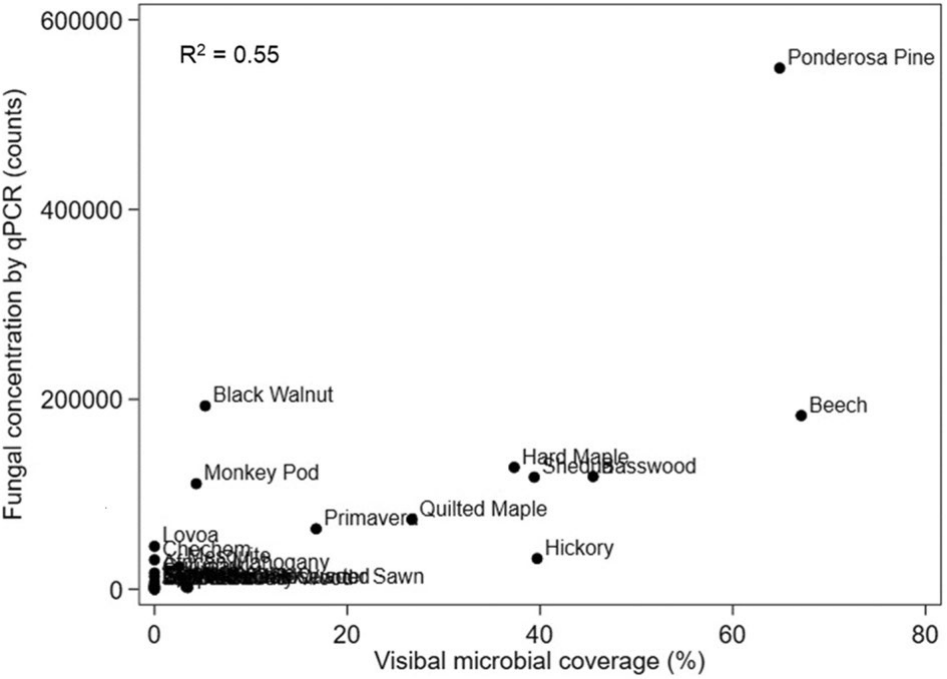
Correlation between visible microbial growth coverage and fungal qPCR concentrations for the 30 tested wood species after 3 weeks of incubation at high RH.

A typical example of visible microbial growth observed from an overhead picture, as well as an example of ImageJ processing to quantify visible growth, is shown in Fig. S1. Visible microbial growth was observed within 3 weeks on 13 of the 30 wood species, with different wood species demonstrating widely varying susceptibility to growth (Fig. 1). Of the 13 species with the greatest visible coverage area, Beech (*Fagus grandifolia*) showed the greatest amount of coverage followed by Ponderosa Pine (*Pinus ponderosa*) and Basswood (*Tilia americana*). Similarly, for fungal qPCR outcomes, we observed that Ponderosa Pine had the greatest fungal abundance, followed by Beech, Black Walnut (*Juglans nigra*), and Hard Maple (*Acer saccharum*) (Fig. 2). The correlation between visible microbial growth coverage and fungal qPCR concentrations, both at the end of the incubation period, was relatively strong given the uncertainties involved in swabbing, extraction, and PCR reactions (R^2^ = 0.55; Fig. 3).

### Relative abundance of fungal taxa

**Figure 4:**
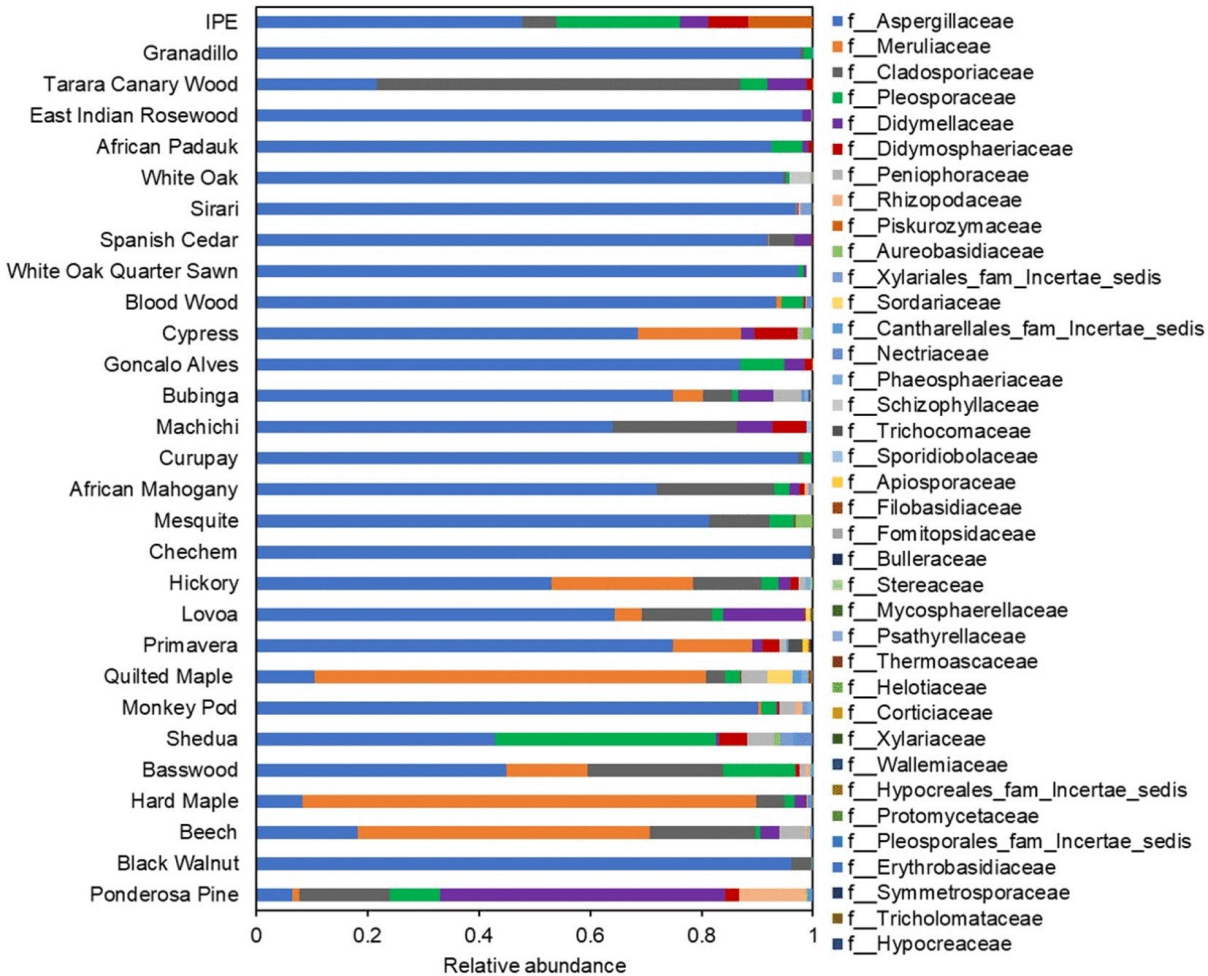
Fungal relative abundance detected by ITS sequencing in 30 different wood materials (with the exception of Leopardwood, which did not yield any ITS DNA) after 3 weeks of high RH exposure. Wood species are shown in descending order of qPCR magnitude from bottom to top, and the legend is sorted by taxa prevalence.

Using DADA2 amplicon inference software^45^, 274 amplicon sequence variants (ASVs) were inferred from the ITS amplicon data. These ASVs represent the overall distinct fungal community members identified by the ITS sequencing and Fig. S2 shows the rarefaction curve for each wood type. The ASVs most likely taxonomy classification was determined by mapping their ITS nucleotide sequences to the UNITE reference database^46^. These taxonomies were later grouped at the family level for each wood type to identify the specific signatures and patterns across different materials (Fig. 4). Wood species are shown in descending order of qPCR magnitude from bottom to top, and the legend is sorted by taxa prevalence. Despite all being naturally inoculated in the same environment and wetted with the same water, it appears that inherent differences in material composition contributed to differences in which fungal families thrived after wetting and being held at high RH. For example, *Aspergillaceae* was most abundant across all of the wood samples. However, *Meruliaceae* was more prevalent on the 8 materials that were observed to have the greatest visible microbial growth coverage, especially for Beech and Maple. Ponderosa Pine, which had both high visible microbial growth coverage and high fungal qPCR concentrations, was dominated by *Didymellaceae*, while *Pleosporaceae* appeared in high abundance only on Shedua. Among the identified families in Fig. 4, *Aspergillaceae, Cladosporiaceae, Tricholomataceae, Sporidiobolaceae* and *Nectriaceae* were also found commonly in homes with dampness and mold^27,47–50^.

### Untargeted metabolomics of microbial and material samples

**Figure 5:**
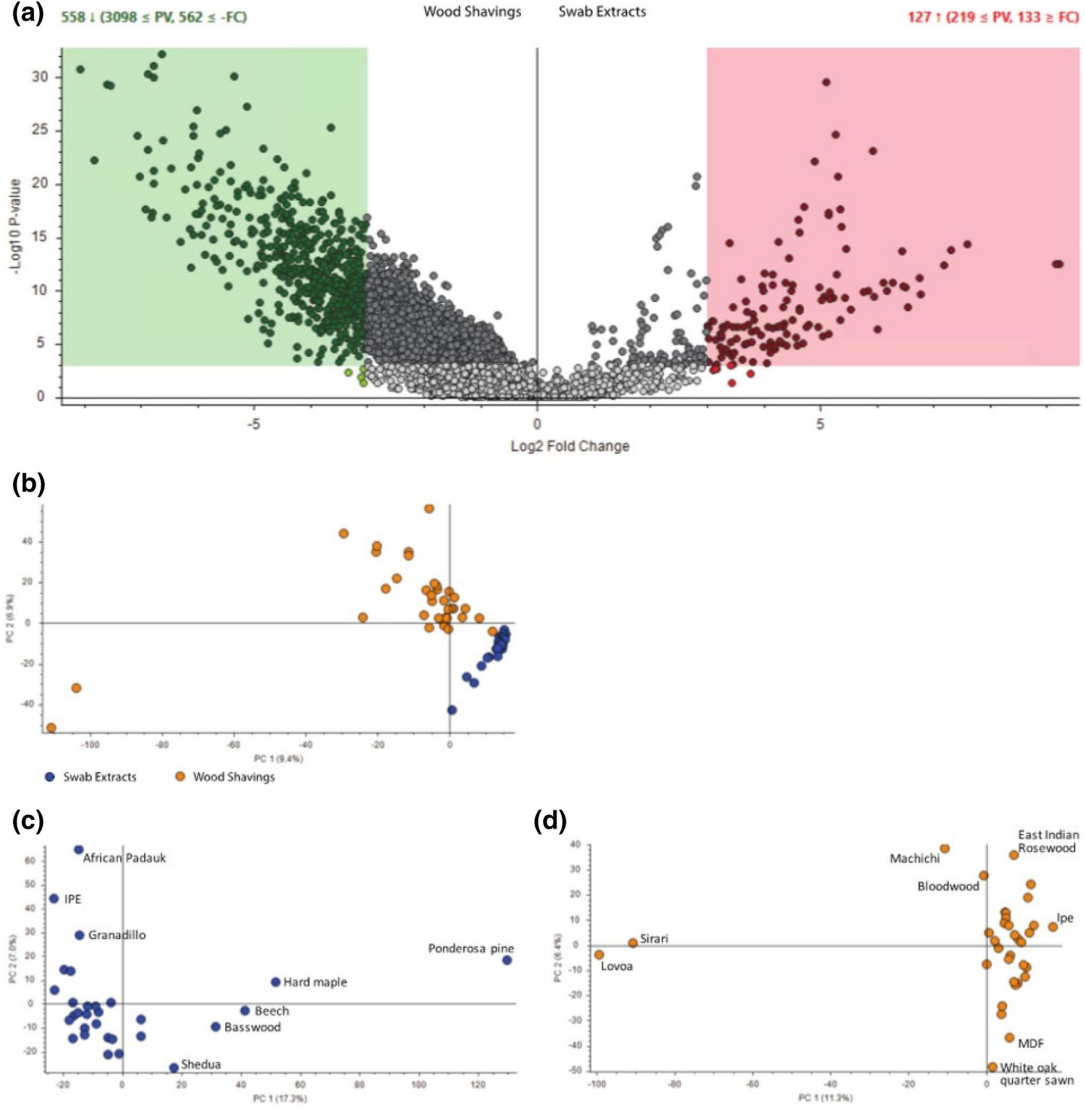
Metabolomics analysis of microbial swab extracts and wood shavings: (**a**) Volcano plot indicating metabolites that are selectively present in either wood shaving samples (left, green dots) or in swab extract samples (right, red dots), (**b**) PCA plot contrasting clustering of swab extract samples (blue) and wood shavings samples (gold), (**c**) PCA plot for swab extract samples only, and (**d**) PCA plot for wood shaving samples only.

Figure 5a shows a comparison of the microbial and wood metabolites in a volcano plot, which revealed several broad trends. Although there is some overlap in the metabolites detected in these two data sets, there is a large number of metabolites that significantly differentiate the two groups, with differences in relative abundance up to 500-fold. Additionally, a larger and more diverse set of metabolites was detected from the wood shavings compared to the microbial swabs, indicating a complex and heterogeneous chemical composition within the wood types.

Data visualization by principal component analysis (PCA) plots revealed further insights. Comparison of the wood shavings and swab extracts on the same plot (Fig. 5b) shows that these groups have distinct signatures, and also that the swab extract samples cluster tightly together, indicating low heterogeneity. These observations mirror the trends seen in Fig. 5a. Furthermore, PCA of either the wood shaving or swab extract samples on their own demonstrates that metabolomics data can be used to distinguish between different wood types. For example, within the swab extract samples (Fig. 5c), Ponderosa Pine was significantly separated from the other wood types; interestingly, this material also exhibited one of the highest levels of fungal growth (Figs. 2, 3) as well as a unique signature of fungal taxa (Fig. 4). Within the wood shaving samples, Sirari and Lovoa wood samples separate significantly from the others, across the PC1 dimension (Fig. 5d). Both of these wood types showed very low levels of microbial growth.

### Chemical composition of wood material extractives and associations with microbial growth

**Figure 6:**
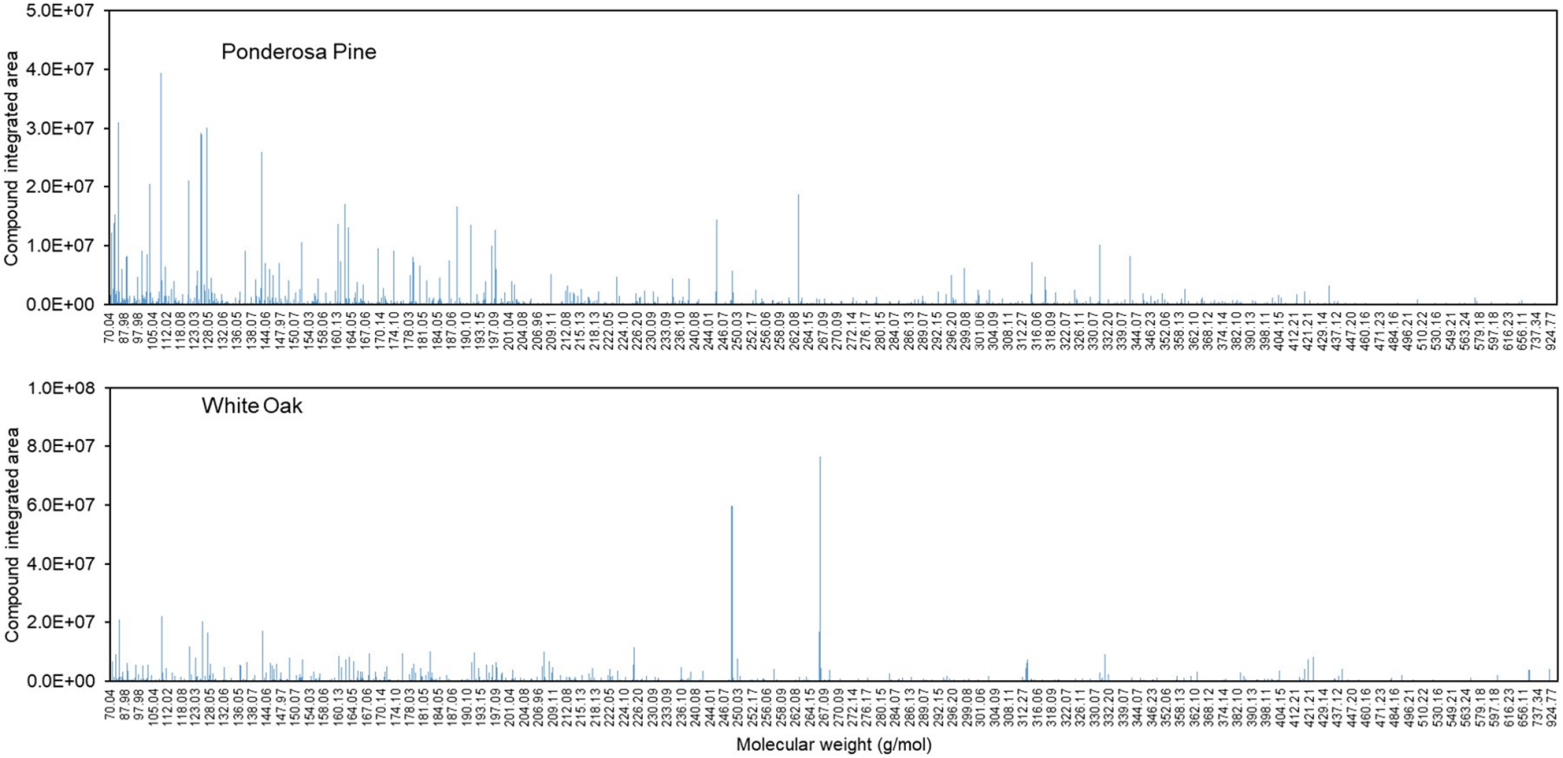
Chemical compounds identified (represented by molecular weight) and quantified (represented by compound integrated area) in Ponderosa Pine and White Oak wood shavings.

From the metabolomics analysis of wood shaving samples, a total of 5,375 spectral features were correlated with molecular formulas. Examples of chemical composition results for both Ponderosa Pine and White Oak are shown in Fig. 6 for illustration. Spectra data from the other 28 wood samples are also shown in Fig. S3.

#### Multi-compound analysis

**Figure 7:**
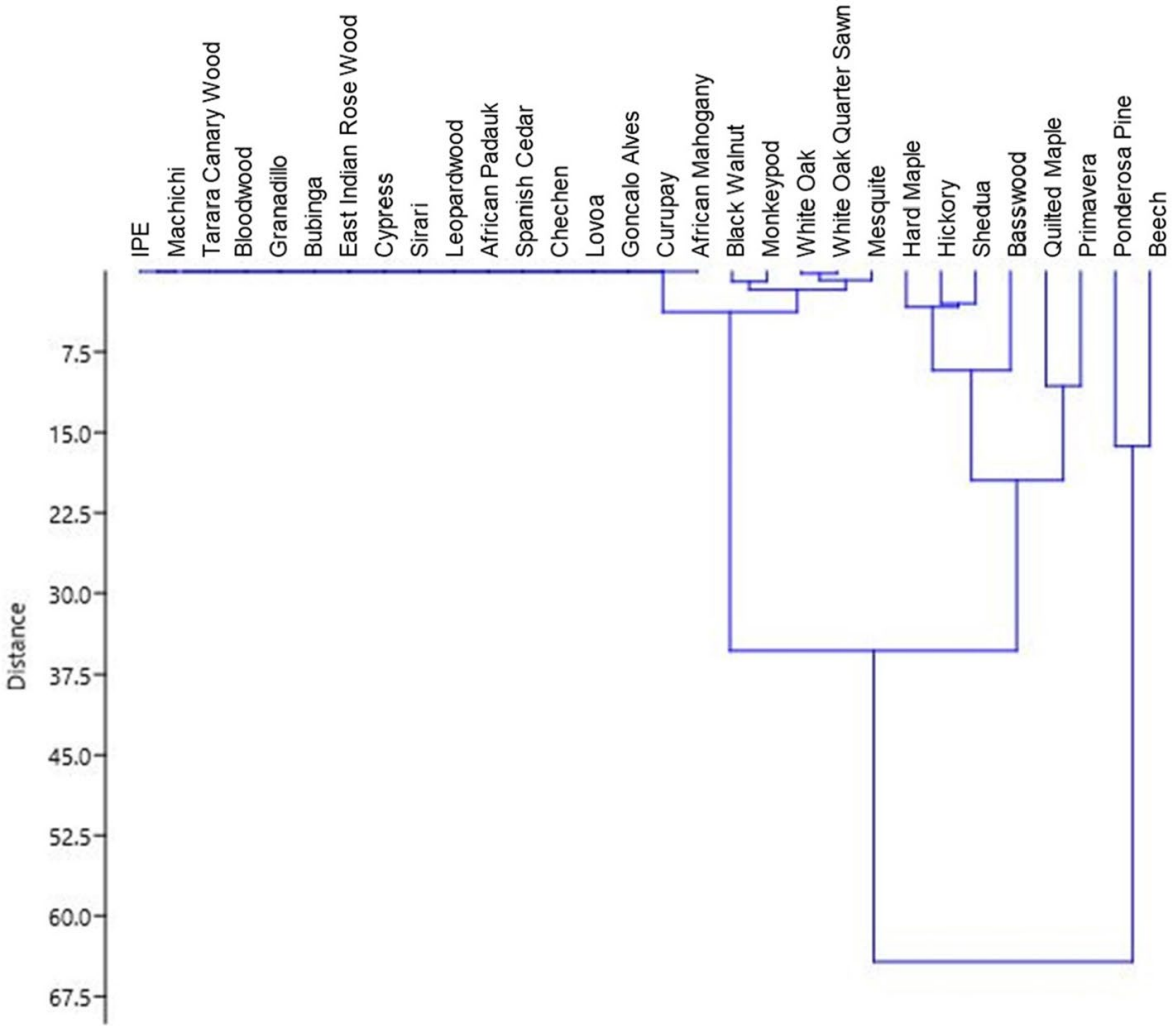
Clustering analysis of 30 wood materials by microbial growth.

Figure 7 and Table 1 show results from the ANOSIM cluster analysis to explore the potential for clusters of multiple compounds to be associated with microbial growth. Results from this dissimilarity test suggest that multiple compound composition factors had only small effects on the microbial growth variables (average ANOSIM R ≈ 0.2) with borderline significance (*p* ranged from 0.05 to 0.07). This cursory analysis suggests that there were no particular suites of compounds that were clearly associated with the extent of microbial growth coverage, although the borderline level of significance suggests similar approaches are warranted in future studies.

**Table 1:** Dissimilarity coefficients between groups (group 1: heavy growth; group 2: light growth; group 1: no growth).

	Group 1 versus Group 2	Group 1 versus Group 3	Group 2 versus Group 3
R	0.23	0.18	0.24
P	0.05	0.05	0.07

#### Single-compound analysis

Tables 2 and 3 show the top 35 and 25 compounds identified and quantified that were most strongly correlated with visible microbial growth and fungal growth quantified via qPCR, respectively (e.g., Spearman correlation coefficients > 0.6 and *p* < 0.001). Compound molecular weights and speculated compound formulas are both shown; note that compound formulas are speculative because compounds identified with a particular molecular weight could represent different compounds depending on molecular structure. Also noted in Tables 2 and 3 are those compounds for which correlations were strongly correlated with both microbial growth outcomes, as well as those compounds that showed the clearest monotonic relationships between abundance and microbial growth outcome upon visual inspection (which is approximately 10 compounds per outcome). Correlations are also shown visually in Fig. S4 and S5 for the same 25–35 speculatively identified compounds.

**Table 2:** Top 35 compounds that showed the strongest Spearman rank correlations with visible microbial growth.

Compound formula	MW (g/mol)	Spearman rho	*p* value
C_11_H_16_O_4_*	212.10	0.74	2.71E−06
C_15_H_12_O_5_	272.07	− 0.74	2.90E−06
C_6_H_7_N_2_O_6_P	234.01	− 0.72	8.17E−06
C_18_H_28_O_4_*^†^	308.20	0.72	8.88E−06
C_10_H_14_O_3_*	182.09	0.71	1.07E−05
C_12_H_20_O_5_	244.13	0.71	1.33E−05
C_12_H_22_O_6_	262.14	0.70	1.76E−05
C_12_H_16_N_2_O_12_*^†^	380.07	0.70	1.79E−05
C_25_H_20_N_4_O_4_*	440.15	0.69	2.62E−05
C_32_H_36_O_11_	596.23	0.68	3.15E−05
C_32_H_32_N_4_O_7_	584.22	0.68	3.92E−05
C_6_H_13_N*	99.10	− 0.67	4.51E−05
C_6_H_15_NO*^†^	181.09	− 0.66	6.38E−05
C_10_H_16_N_6_O_4_	284.12	0.66	7.94E−05
C_32_H_36_O_12_	612.22	0.65	9.20E−05
C_8_H_10_O_3_*	154.06	0.65	1.03E−04
C_12_H_20_O_4_	228.14	0.65	1.07E−04
C_19_H_22_O_5_	330.15	0.65	1.07E−04
C_10_H_10_N_4_O_2_S	250.05	− 0.65	1.08E−04
C_20_H_28_O_3_	316.20	0.64	1.29E−04
C_49_H_100_N_5_O_11_PS_3_	1,061.63	− 0.64	1.35E−04
C_13_H_8_O_5_	244.04	− 0.64	1.44E−04
C_18_H_35_NO_4_	329.26	0.64	1.51E−04
C_12_H_16_O_5_	240.10	0.63	1.70E−04
C_10_H_22_N_2_O_6_	266.15	0.63	1.76E−04
C_17_H_14_O_4_	282.09	− 0.63	1.76E−04
C_12_H_23_N_4_O_6_PS*	382.11	0.63	1.78E−04
C_5_H_13_NO	103.10	0.63	1.84E−04
C_15_H_14_O_5_	274.08	− 0.63	1.87E−04
C_17_H_16_O_6_	316.09	− 0.63	1.87E−04
C_7_H_6_O_2_	122.04	− 0.63	1.95E−04
C_3_H_4_N_3_O_5_P_3_	254.94	− 0.63	2.07E−04
C_20_H_32_O_4_*	353.26	0.63	2.17E−04
C_11_H_14_O_4_	210.09	0.62	2.27E−04
C_18_H_30_O_3_	294.22	0.62	2.37E−04

*Promising compounds with near-monotonic relationships with visible fungal growth.

*^†^Promising compounds that correlate with both fungal growth outcomes.

**Table 3:** Top 25 compounds that showed the strongest Spearman rank correlations with fungal qPCR concentrations.

Compound formula	MW (g/mol)	Spearman rho	*p* value
C_18_H_34_O_4_*	314.25	0.77	7.19E−07
C_18_H_35_NO_4_*	329.26	0.74	2.94E−06
C_14_H_12_O_3_	228.08	0.69	2.73E−05
C_18_H_28_O_4_*^†^	308.20	0.68	3.12E−05
C_18_H_30_O_3_*	294.22	0.67	4.76E−05
C_6_H_14_S_3_*	182.03	0.66	6.20E−05
C_6_H_15_NO*^†^	181.09	− 0.66	7.12E−05
C_20_H_26_O_3_	314.19	0.65	1.03E−04
C_12_H_16_N_2_O_12_*^†^	380.07	0.65	1.10E−04
C_15_H_12_O_4_	256.07	− 0.63	2.11E−04
C_5_H_8_O*	84.06	0.63	2.14E−04
C_16_H_16_O_5_	288.10	− 0.63	2.20E−04
C_12_H_23_NO_4_	245.16	0.62	2.23E−04
C_16_H_17_N_5_O_4_P_2_	405.08	0.62	2.45E−04
C_11_H_9_NO_2_*	187.06	0.62	2.79E−04
C_19_H_14_N_2_O_5_S	382.06	0.62	2.83E−04
C_20_H_28_O_3_*	316.20	0.61	3.26E−04
C_12_H_6_N_6_O	250.06	0.61	3.26E−04
C_8_H_8_O	120.06	− 0.60	4.24E−04
C_18_H_28_O_2_*	276.21	0.60	4.30E−04
C_4_H_6_N_6_O_4_	202.05	0.60	4.46E−04
C_18_H_30_O_2_	278.22	0.60	4.52E−04
C_10_H_15_NO_5_	229.09	0.60	4.57E−04
C_13_H_18_O_9_	318.09	0.60	4.98 E−04
C_9_H_12_N_2_O_3_S_2_	260.03	0.60	5.04E−04

*Promising compounds with near-monotonic relationships with fungal qPCR concentration.

*^†^Promising compounds that correlate with both fungal growth outcomes.

**Figure 8:**
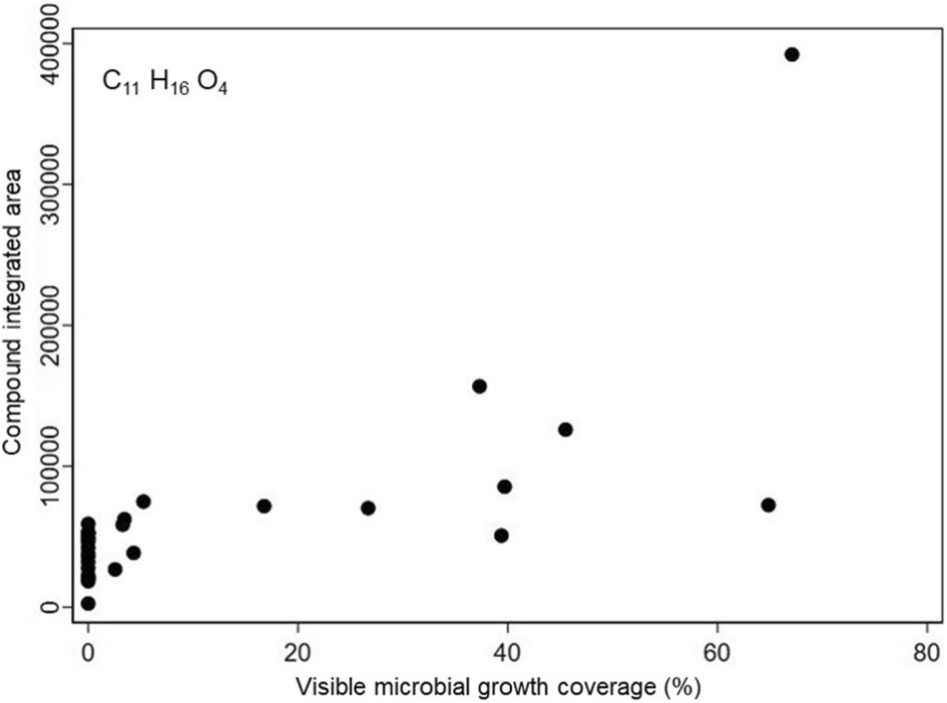
Abundance of C_11_H_16_O_4_ versus visible microbial growth percentage at the end of the 3-week test (Spearman rho = 0.742).

**Figure 9:**
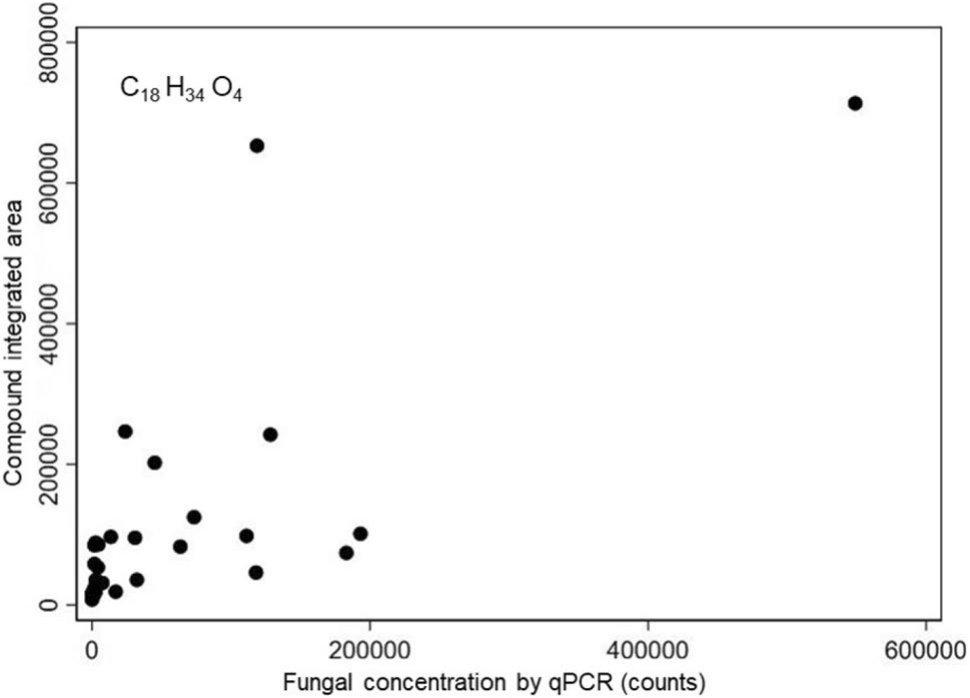
Abundance of C_18_H_34_O_4_ versus fungal qPCR concentration at the end of the 3-week test (Spearman rho = 0.768).

Results indicate that the putatively identified C_11_H_16_O_4_ had the strongest correlation with visible microbial growth, with a Spearman’s rank correlation coefficient of 0.742 (*p* < 0.0001) (Fig. 8). The compound formula that was most strongly correlated with fungal qPCR concentration was C_18_H_34_O_4_, with a Spearman’s rank correlation coefficient of 0.768 (*p* < 0.0001) (Fig. 9). A strong positive correlation in these analyses suggest the potential for the presence/abundance of these compounds may encourage fungal proliferation.

**Figure 10:**
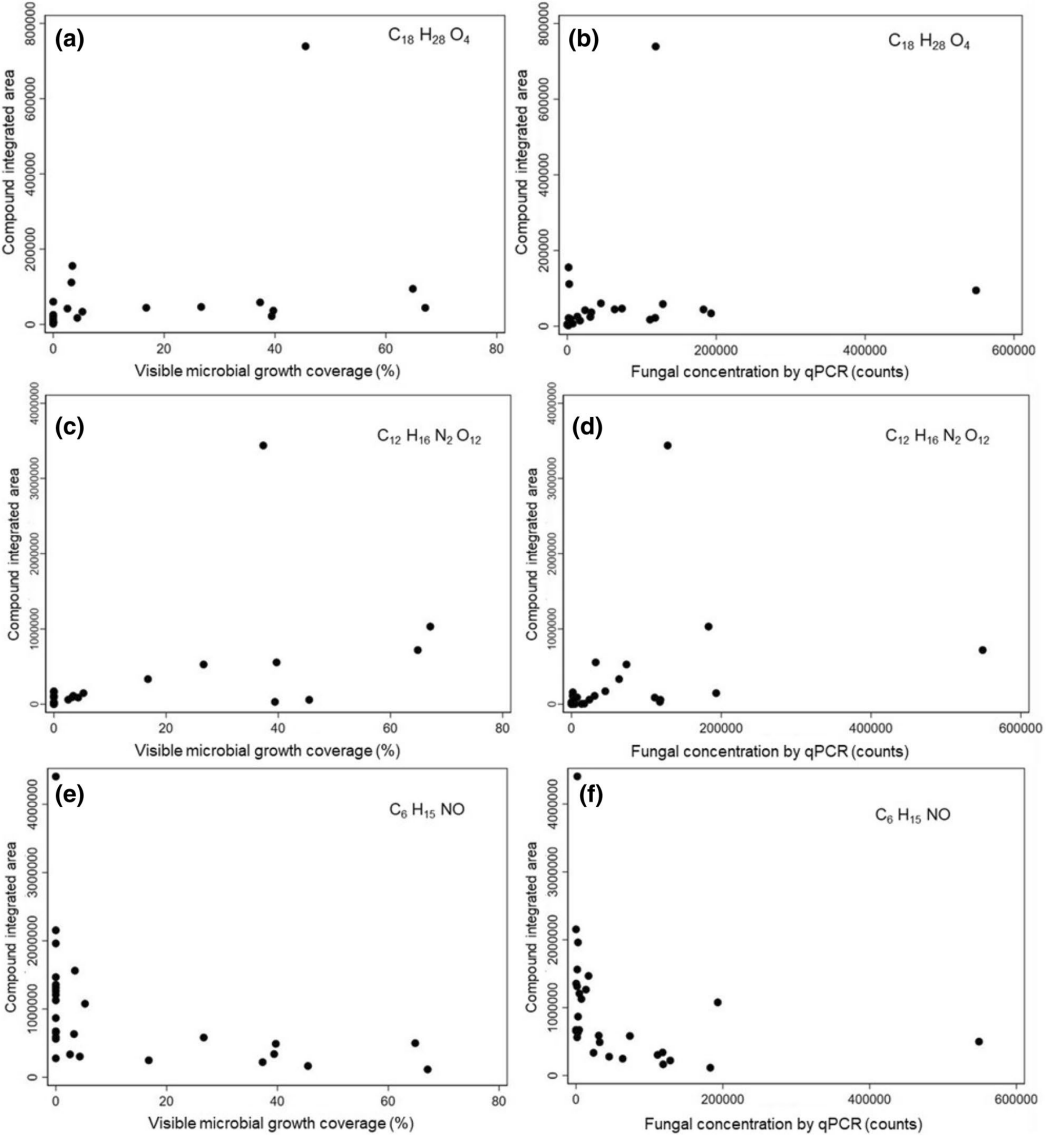
Abundance of three putatively identified chemical compounds versus microbial growth outcomes at the end of the 3-week test: (**a**) C_18_H_28_O_4_ versus visible growth, (**b**) C_18_H_28_O_4_ versus fungal qPCR, (**c**) C_12_H_16_N_2_O_12_ versus visible growth, (**d**) C_12_H_16_N_2_O_12_ versus fungal qPCR, (**e**) C_6_H_15_NO versus visible growth, and (**f**) C_6_H_15_NO versus fungal qPCR.

Figure 10 shows compound abundance versus microbial growth for several of the identified chemical compounds that were strongly correlated with both visible microbial growth and qPCR fungal growth outcomes. For example, C_18_H_28_O_4_ revealed a strong and significant correlation with visible growth (rho = 0.72, *P* < 0.0001) and fungal qPCR concentrations (rho = 0.68, *P* < 0.0001), although the relationship appears largely driven by a small number of outliers (Fig. 10a, b). C_12_ H_16_ N_2_ O_12_ was strongly correlated with both outcomes, although little information could be found about the potential names of this compound formula (Fig. 10c, d). The compound formula C_6_H_15_NO had a strong negative correlation with both microbial growth outcomes (rho = 0.66, *P* < 0.0001), with a shape that suggests a near-monotonic nonlinear response below a particular threshold for both growth outcomes (Fig. 10e, f). Because of these combined factors, we conducted a pilot experiment to investigate the inhibitory effects of dosing a sample of wood materials (Ponderosa Pine) with varying concentrations of pure liquid C_6_H_15_NO (assuming it was present in wood samples as Diethylethanolamine) mixed with both tap water and distilled water at varying concentrations.

### Inhibitory effect of C_6_H_15_NO on visible microbial growth assessment

**Figure 11:**
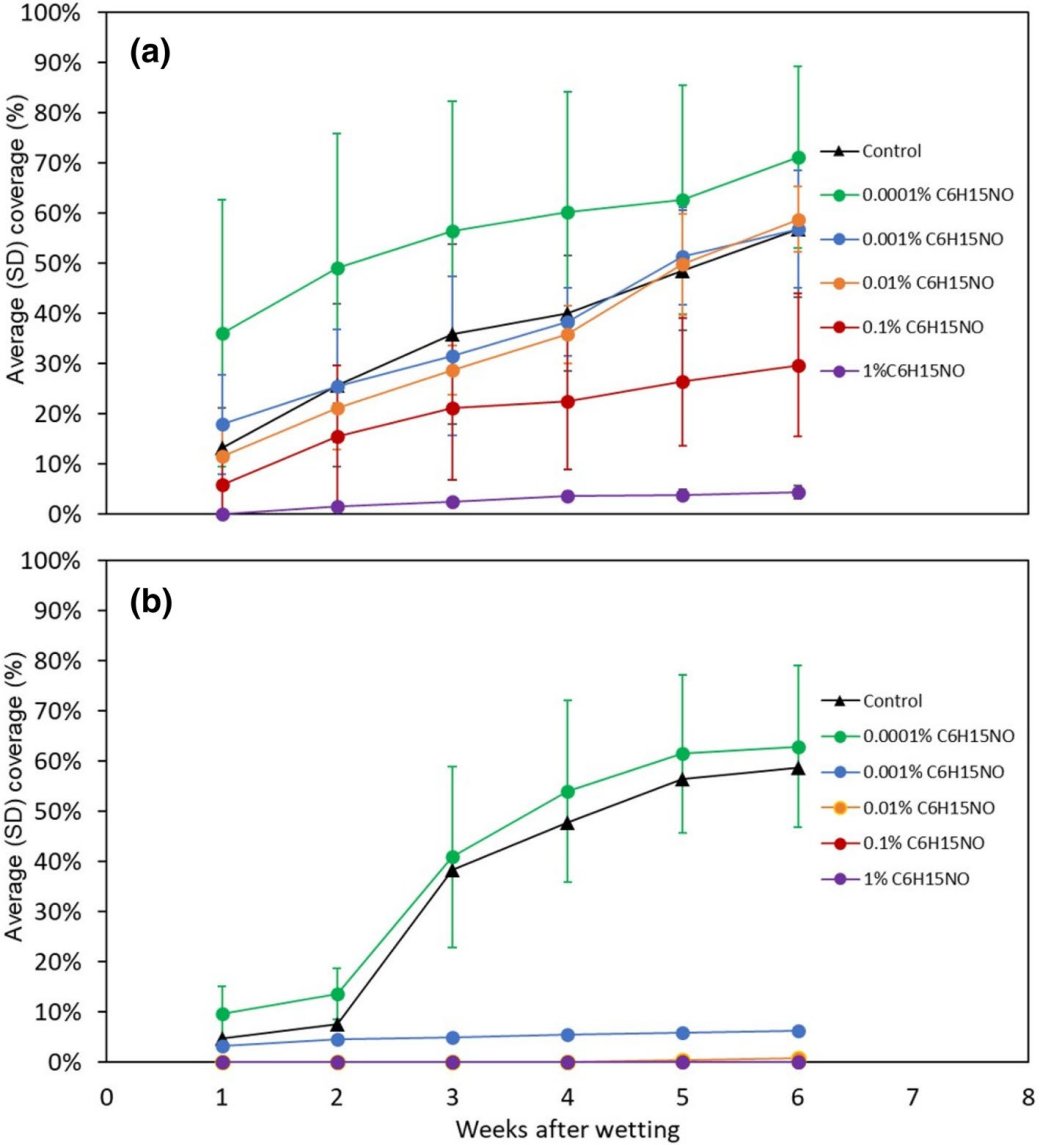
Visible microbial growth coverage over time for Ponderosa Pine samples wetted with solutions with varying concentrations of C_6_H_15_NO: (**a**) tap water and (**b**) distilled water.

Figure 11 shows the mean (± S.D.) visible microbial growth coverage areas on the Ponderosa Pine samples over time after wetted with varying concentrations of C_6_H_15_NO (assuming Diethylethanolamine) solutions mixed with (a) tap water and (b) distilled water. Test coupons that were wetted with tap water and the lowest concentration (0.0001%) of C_6_H_15_NO showed the greatest amount of microbial coverage, ranging from ~ 36% for the first week to ~ 71% for the last week (Fig. 11a). Conversely, coupons that were wetted with tap water and the greatest concentration (1%) of C_6_H_15_NO showed the lowest amount of microbial coverage, with only ~ 4% at the last week of incubation (Fig. 11a). The amount of microbial coverage area on the coupons of the tap water control group was ~ 13% for the first week and increased to ~ 57% for the 6th week, which is similar in magnitude to the coupons that were wetted with 0.001% and 0.01% solutions of C_6_H_15_NO.

Similarly, the coupons that were wetted by distilled water solutions mixed with C_6_H_15_NO showed less overall microbial growth compared to the coupons wetted with tap water (Fig. 11b). However, the inhibitory effect of C_6_H_15_NO is still clearly demonstrated, as the distilled water control group and lowest concentration of C_6_H_15_NO (0.0001%) showed the greatest visible microbial growth, followed by small amounts of visible growth at 0.001% C_6_H_15_NO and negligible or no visible growth at 0.01% C_6_H_15_NO and higher.

## Discussion

Results of this study indicate that the extractive chemical composition of the tested wood material samples has a significant effect on the magnitude and dynamics of microbial growth on wetted surfaces. Moreover, results suggest that a better understanding of fungal colonization susceptibility of different materials commonly used in construction could be used to limit adverse health outcomes caused by exposure to pathogenic fungal species and lessen the chance of fungal-associated wood rot.

The identified fungal families are mainly composed of saprobic species that decompose and digest plant matter. However, several contain members that are mycoparasitic or pathogenic, either to plants or animals. The fungal families *Aspergillaceae, Aureobasidiaceae, Cladosporiaceae,* and *Nectriaceae* all contain one or more pathogenic species that can cause disease in humans. The pathogenic species from these fungal families can all be characterized as opportunistic pathogens, most often infecting those with weakened immune systems such as cancer patients, people with disorders of their immune systems, or those taking drugs that intentionally suppress the immune system^51–53^.

Invasive aspergillosis, caused by certain species of the *Aspergillaceae* family, is a disease that can occur in several organs of the body but is most often associated with pulmonary infections initiated by the inhalation of fungal spores^54–57^. A devastating illness for immunocompromised patients, mortality rates linked to invasive aspergillosis can range from 40 to 90% in certain cases^58^. Chronic human exposure to one species of *Aureobasidiaceae*, *Aureobasidium pullulans*, can induce hypersensitivity pneumonitis^59,60^. Commonly referred to as “humidifier lung”, this disease is characterized by maladies of the lungs, including coughing, dyspnea, and acute inflammation. Species from the *Cladosporiaceae* family, although rarely pathogenic to humans, produce airborne spores that if one is exposed to over an extended period can cause adverse health effects, especially for people with asthma or those suffering from other respiratory diseases^61,62^. For *Nectriaceae,* the majority of species from this family are innocuous, soil-borne saprobes but several have been reported as opportunistic pathogens while others produce harmful mycotoxins^63^.

The fungal families *Fomitopsidaceae*, *Merulicaeae*, and *Schizophyllaceae* all cause forms of wood rot^64^. Brown rot, associated with members of *Fomitopsidaceae,* results from the breakdown of the structural compounds cellulose and hemicellulose by fungal enzymes and alters the wood into shrunken, brown-discolored cubical pieces^65^. Differing from *Fomitopsidaceae,* certain species of *Meruliaceae* and *Schizophyllaceae* families cause another form of wood rot called white rot, wherein the lignin of moist wood is broken down, leaving behind a stringy, light-colored material composed mostly of undigested cellulose^66,67^.

Of the individual chemical compounds that were most strongly correlated with the extent of visible microbial growth and/or fungal qPCR concentrations, several may have some plausible explanations. For example, possible names for the putatively identified C_11_H_16_O_4_, which was positively correlated with visible growth, could be methylenolactocin and DETOSU. According to a compound search, methylenolactocin is apparently known as an isolate of Penicillium with anti-cancer activity. Similarly, a possible name for the putatively identified C_6_H_15_NO, which had a strong negative correlation with both microbial growth outcomes, includes Diethylethanolamine, which is used as a corrosion inhibitor and a precursor in the production of a local anesthetic. Subsequent testing of the application of this individual compound at varying concentrations in tap water confirmed its inhibitory effects. Given that the compound is an alcohol with some known toxicity, the association is not particularly surprising, and the compound may not be a viable candidate for use as an anti-fungal agent in materials. However, the process used herein to identify individual and/or clusters of compounds intrinsic to materials that correlate with microbial growth outcomes can be utilized and expanded to isolate other candidate compounds from woods and other material and could potentially inform their integration into other types of materials to increase microbial growth resistance under wetting conditions.

While these data provide novel insights into the chemical drivers of differential microbial growth susceptibility within this otherwise homogenous set of material samples, it is important to note several limitations to this work, including: (1) only one test coupon for each type of wood was used, which does not allow for capturing variability inherent in microbial dynamics and growth on surfaces of the same material composition; (2) the material chemical composition analysis targeted a specific range of compounds (i.e., 70–1,050 m/z); other compositional analysis approaches could uncover additional insights outside of these bounds; and (3) the underlying mechanisms explaining observed associations between material compositions and microbial growth are not explored in detail. Future work with other materials and analytical approaches should focus on overcoming these limitations.

## Methods

Thirty (30) different wood species were selected to study microbial growth susceptibility and community structure upon wetting. The wood samples were all purchased new in a kit from an online retailer in an attempt to collect a wide variety of wood species that were likely to experience a diversity of microbial growth patterns upon wetting. The wood samples were naturally seeded with environmental microbes, wetted, and then incubated at high relative humidity (RH) conditions. Microbial growth was assessed over time using a combination of visual assessment and quantitative polymerase chain reaction (qPCR). The same samples were also swabbed at the end of the experiments for ITS (fungal) rRNA amplicon sequencing. Additionally, powders from unwetted duplicates of each type of wood were shaved and collected for material chemical composition analysis using reverse phase liquid chromatography with tandem mass spectrometry (RPLC-MS-MS).

### Preparation of materials

The names and categorical classification of rot resistance of each wood type utilized are listed in Table 4 68–70. All tested wood materials were cut to 5 cm × 7.5 cm coupons and sterilized by UV. The samples were then naturally inoculated by environmental microbes by leaving the sterilized coupons unprotected in a laboratory setting for ~ 30 days. Next, to simulate what happens after a material comes in direct contact with bulk liquid from a flood or leak, all 30 wood coupons were submerged in tap water for ~ 12 h. Tap water (as compared to distilled water) was chosen to provide more realistic growth from a wetting event. The same laboratory tap water source was used to wet all coupons at the same time. After wetting, each coupon was then placed in individual petri dishes and incubated at room temperature (20–25 °C) inside a static airtight chamber at high RH for ~ 3 weeks to encourage fungal growth. Potassium nitrate salt solutions were used to maintain RH at ~ 94% for the duration of the experiment.

**Table 4.** 30 types of woods selected for testing.

	Name	Scientific name	Type	Rot resistance^68–70^
1	Beech	*Fagus grandifolia*	Hardwood	Slightly or nonresistant
2	Basswood	*Tilia americana*	Hardwood	Slightly or nonresistant
3	Hickory	*Carya ovata*	Hardwood	Slightly or nonresistant
4	Quilted Maple	*N/A*	Hardwood	Slightly or nonresistant
5	Hard Maple	*Acer saccharum*	Hardwood	Slightly or nonresistant
6	African Mahogany*	*Khaya spp.*	Hardwood	Moderately resistant
7	Bubinga*	*Guibourtia spp.*	Hardwood	Moderately resistant
8	Lovoa*	*Lovoa trichilioides*	Hardwood	Moderately resistant
9	Ponderosa Pine	*Pinus ponderosa*	Softwood	Moderately resistant
10	Shedua*	*Guibourtia ehie*	Hardwood	Moderately resistant
11	Primavera	*Roseodendron donnell-smithii*	Hardwood	Moderately resistant
12	Cypress	*Taxodium distichum*	Softwood	Resistant
13	Leopardwood	*Roupala montana*	Hardwood	Resistant
14	Mesquite	*Prosopis glandulosa*	Hardwood	Resistant
15	Spanish Cedar*	*Cedrela odorata*	Hardwood	Resistant
16	White Oak	*Quercus alba*	Hardwood	Resistant
17	White Oak Quarter Sawn	*Quercus alba*	Hardwood	Resistant
18	Walnut Black	*Juglans nigra*	Hardwood	Resistant
19	African Padauk*	*Pterocarpus soyauxii*	Hardwood	Very resistant
20	Bloodwood	*Brosimum rubescens*	Hardwood	Very resistant
21	Chechen*	*Metopium brownei*	Hardwood	Very resistant
22	Curupay*	*Anadenanthera colubrina*	Hardwood	Very resistant
23	East Indian Rosewood*	*Dalbergia latifolia*	Hardwood	Very resistant
24	Granadillo	*Platymiscium spp.*	Hardwood	Very resistant
25	Goncalo Alves	*Astronium spp.*	Hardwood	Very resistant
26	IPE*	*Handroanthus spp.*	Hardwood	Very resistant
27	Monkeypod	*Albizia saman*	Hardwood	Very resistant
28	Machiche	*Lonchocarpus spp.*	Hardwood	Very resistant
29	Sirari	*Guibourtia hymenaeifolia*	Hardwood	Very resistant
30	Tarara Canary Wood	*Centrolobium spp.*	Hardwood	Very resistant

*Imported wood as noted on the retailer’s website.

### Microbial growth assessment

Microbial growth was assessed using multiple methods. Visual microbial growth was assessed by taking overhead images on a weekly basis during a ~ 3-week incubation period. Image analyses were conducted using ImageJ to estimate the percentage of microbial growth coverage over time using the area fraction option. Additionally, at the end of the incubation period, the surfaces of the coupons were swabbed using sterile polyester swabs for subsequent sequencing and analysis, including ITS for fungal communities and qPCR for fungal quantification using universal primers. Finally, coupon surfaces were also swabbed with cotton-tipped swabs that were dipped in ethanol for subsequent surface chemistry and metabolomics analysis.

### DNA extraction, sequencing, and qPCR

The microbial community from each sample was collected by rubbing the tips of sterile polyester swabs along the surface of the coupons. Following sample collection, the swab tips were cut off into DNA extraction tubes (DNeasy Powersoil, Qiagen), and the DNA extracted following the manufacturer’s protocol^71^ with the following modification to reduce sample loss: combine steps 7 through 10 by adding 150 µL each of solutions C2 and C3 to the tube that the lysed sample was transferred to in step 6. Following a 5-min incubation period at 4 °C, the manufacturer’s protocol is resumed as normal at step 11.

Amplification of the ITS region used the Illumina Earth Microbiome ITS protocol^72^. Reactions were pooled, cleaned with Agencourt AMPure beads, and then the clean amplicon pool was sequenced at Argonne National Laboratory’s Environmental Sample Preparation and Sequencing Facility, following the Earth Microbiome Protocol^73^. For PCR cycling the following reaction mix was used: 9.5 μL of molecular biology grade H2O, 12.5 μL of Accustart II PCR Toughmix, 1 μL each of forward and reverse primers at 5 μM, and 1 μL of sample DNA for a total reaction volume of 25 μL. The following PCR program was used: Initial denaturing step at 94 °C for 3 min, followed by 35 cycles of: 94 °C for 45 s, 50 °C for 60 s, and 72 °C for 90 s, followed by a final extension step of 72 °C for 10 min. Sequencing was performed on an Illumina Miseq using V3 chemistry. Fungal amplicons were sequenced using 2 × 300 nt reads.

To quantify the total abundance of fungi, qPCR was performed using a Roche Lightcycler 480 and the SYBR Green I Master kit. Primers targeting the ITS1f.-ITS2 priming sites, without the Illumina adapters or barcodes, were used during amplification. To calculate the abundance for the fungal qPCR, the Femto Fungal DNA Quantification Kit from Zymo was used as a standard control.

### Microbial taxonomy identification

ITS Amplicon produced 797,766 reads from 29 of the 30 woods; Leopardwood did not yield any ITS reads. Using DADA2^45^, the reads were clustered and 274 amplicon sequence variants (ASVs) were identified. The closest taxonomy groups were identified by mapping ASVs to the UNITE database^46^.

### Microbial metabolomics and chemical composition analysis

Unwetted samples of each type of wood were shaved into ~ 100 mg of powder using sterile scalpels on the surface. For each sample, 20 mg wood shavings were transferred to an epitube, then 500 μL of 50/50 methanol/water was added, and the sample was vortexed. The mixture was left overnight at room temperature, and on the following day, samples were centrifuged and supernatant was transferred to a fresh epitube. The supernatants were evaporated to dryness and reconstituted in 100 µL LCMS buffer (5% acetonitrile/water + 0.1% formic acid). Ethanol extracts from surface swabs were evaporated to dryness and reconstituted in 200 µL of LCMS buffer. High resolution mass spectrometry analysis was performed according to previously reported procedures^23^. Analysis of LC–MS/MS data from the microbial swab extracts and the wood shavings was performed with Compound Discoverer 3.0 (Thermo Fisher), which identified metabolites as unique spectral features based on a combination of molecular weight and retention time. The predicted compound hits from Compound Discoverer were filtered to include only those compounds confirmed by both intact mass and MS/MS spectral matching via the mzCloud database. Compounds were identified by their molecular weight and quantified using the compound integrated area.

### Statistical analysis

Nonparametric Spearman rank correlations were first used to explore associations between microbial growth and individual compounds identified and quantified in the material shavings. Over 5,000 identified compounds were first ranked by their Spearman correlation coefficients and p-values using Stata version 15 (StataCorp SE, College Station, TX, USA). The top ~ 30 compounds that showed the strongest correlations with microbial/fungal growth outcomes (positive or negative) were then visually inspected, and those with the clearest monotonic relationships were chosen for further analysis.

Additionally, a dissimilarity analysis was conducted using ANOSIM with the entire suite of 5,000 + compounds from the 30 wood species by using PAST (PAleontological Statistics) version 3^74^ to determine if chemical signatures of multiple compounds (instead of just single compounds) were also correlated with microbial growth. In our visible microbial growth dataset, the 30 wood materials were clustered into three groups based on a histogram of visible coverage level (i.e., heavy growth, light growth, and no growth). The ANOSIM statistic, which compares the mean of ranked dissimilarities between groups to the mean of ranked dissimilaristies within groups, was used to determine whether distances between samples of the same growth level were significantly lower than distances between samples of different growth levels.

### Single compound inhibitory effects: pilot experiment

Following the analysis of the 30 wood samples, a single compound found in the material composition analysis that was strongly and monotonically correlated with fungal growth was then tested in a pilot chemical dosing experiment to evaluate the potential inhibitory effect of the compound. A new batch of samples of ponderosa pine wood (one of the tested woods shown to be most susceptible to fungal growth) was purchased and cut into 5 cm × 7.5 cm coupons and sterilized by UV. The coupons were naturally inoculated again by leaving them unprotected in a laboratory setting for ~ 30 days (same as before). Pure liquid chemical of a single putatively identified compound (C_6_ H_15_ NO) was purchased and mixed with both tap water and distilled water at varying concentrations (by volume), including 0.0001%, 0.001%, 0.01%, 0.1%, and 1%, and controls with 0%. Next, wood coupons were wetted by submerging them into each of the prepared varying concentration mixtures (triplicate coupons at each concentration), covered with aluminum foil, and placed inside a biosafety hood to soak overnight (~ 12 h). The coupons, including a control coupon wetted with sterile water and with none of the dosing compound, were then incubated at room temperature inside the same static airtight chamber at high RH conditions for several weeks (same as before). Microbial growth was evaluated visually on a weekly basis during a ~ 6-week incubation period using the same image processing procedures as described previously.

## Supplementary information

Supplementary Information 1.

Supplementary Information 2.

## Data Availability

The datasets generated during the current study are available from the corresponding author on reasonable request.
