# Supplementary material for: Chemical composition of material extractives influences microbial growth and dynamics on wetted wood materials
Source: Sci Rep. 2020 Sep 2;10:14500. doi: 10.1038/s41598-020-71560-3 (PMC7467922; doi:10.1038/s41598-020-71560-3)
Supplement: Supplementary file 1 — Supplementary Information 1. [file 41598_2020_71560_MOESM1_ESM.docx]

Supplementary Information for:

**Chemical composition of material extractives influences microbial growth and dynamics on wetted wood materials**

Dan Zhao^1^, Cesar Cardona^2,3^, Neil Gottel^4^, Valerie J. Winton^5^, Paul M. Thomas^5^, Daniel A. Raba^6^, Scott T. Kelley^7^, Chris Henry^8^, Jack A. Gilbert^4^, Brent Stephens^1*^

^1^Department of Civil, Architectural, and Environmental Engineering, Illinois Institute of Technology, Chicago, IL USA

^2^ Graduate Program in Biophysical Sciences, The University of Chicago, Chicago, IL USA

^3^ Department of Surgery, The University of Chicago, Chicago, IL USA

^4^ Department of Pediatrics, University of California San Diego School of Medicine, San Diego, CA USA

^5^ Proteomics Center of Excellence and Department of Molecular Biosciences, Northwestern University, Evanston, IL USA

^6^ Department of Biology, Illinois Institute of Technology, Chicago, IL USA

^7^ Department of Biology, San Diego State University, San Diego, CA USA

^8^ Mathematics and Computer Science, Argonne National Laboratory, Lemont, IL USA


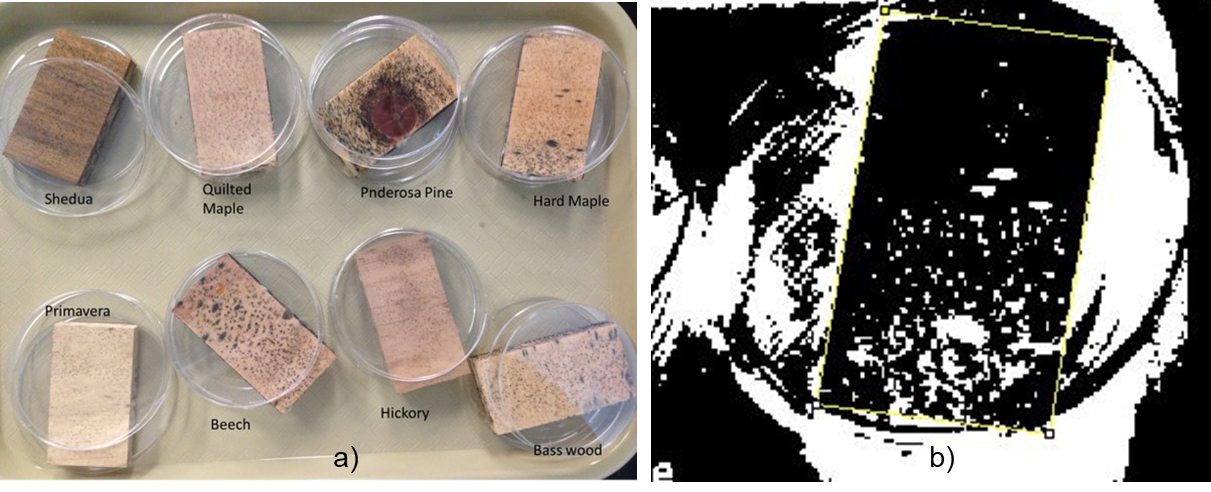


**Figure S1. Example of visible microbial growth: a) Overhead photo of the 8 woods with the heaviest visible microbial growth after the second week of testing; b) ImageJ processed picture of hard maple (the white sections represent visible growth)**


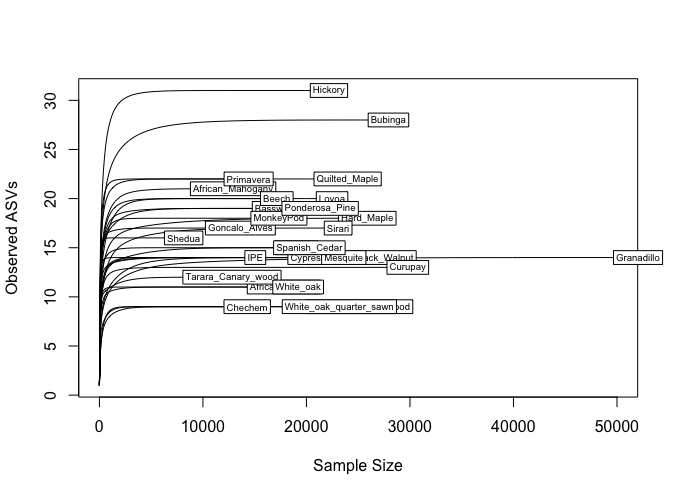


**Figure S2. Rarefaction curves for 30 different woods**


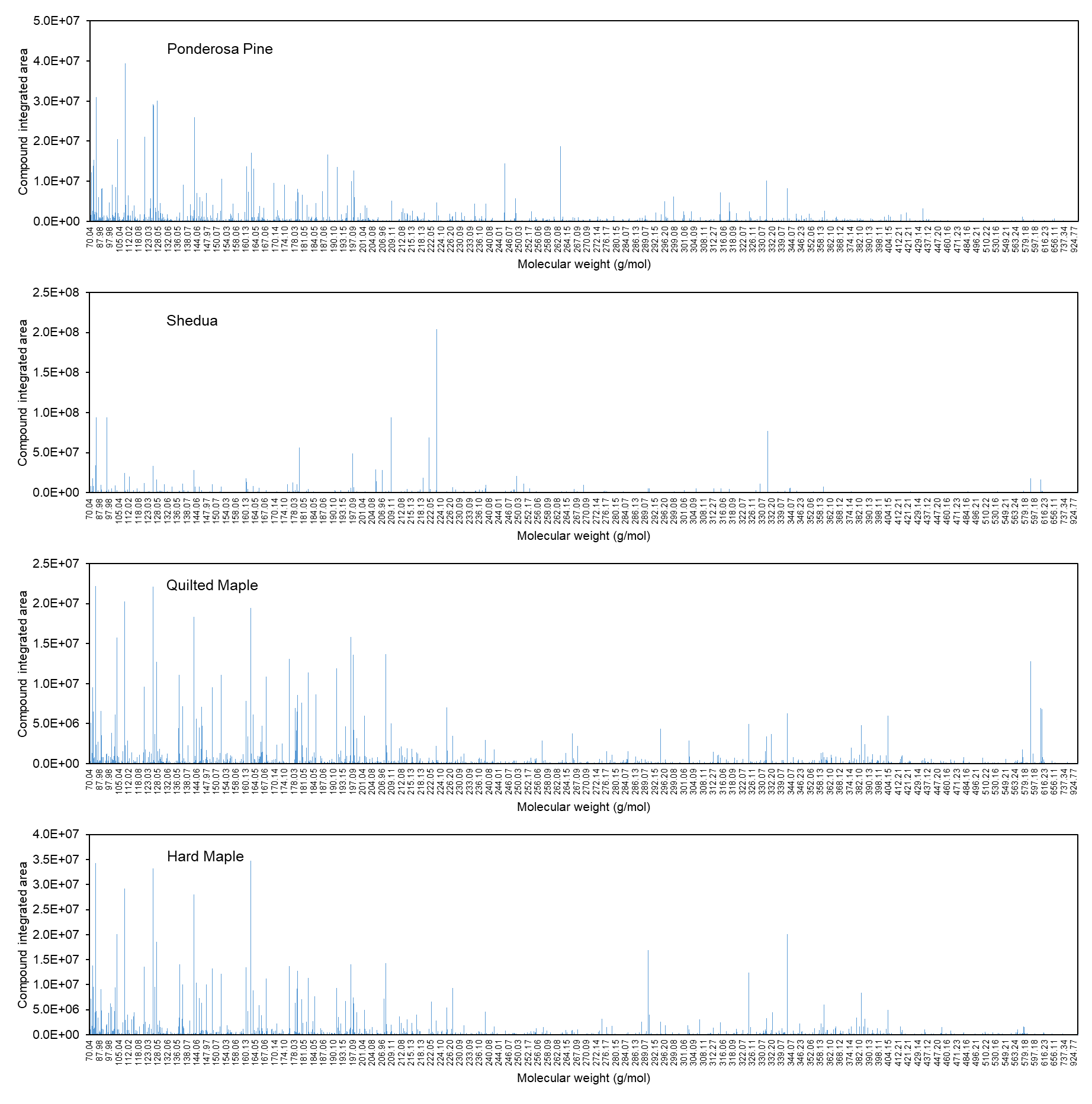


**Figure S3. Chemical compounds (identified by molecular weight and quantified by compound integrated area) in shavings from 30 different woods**


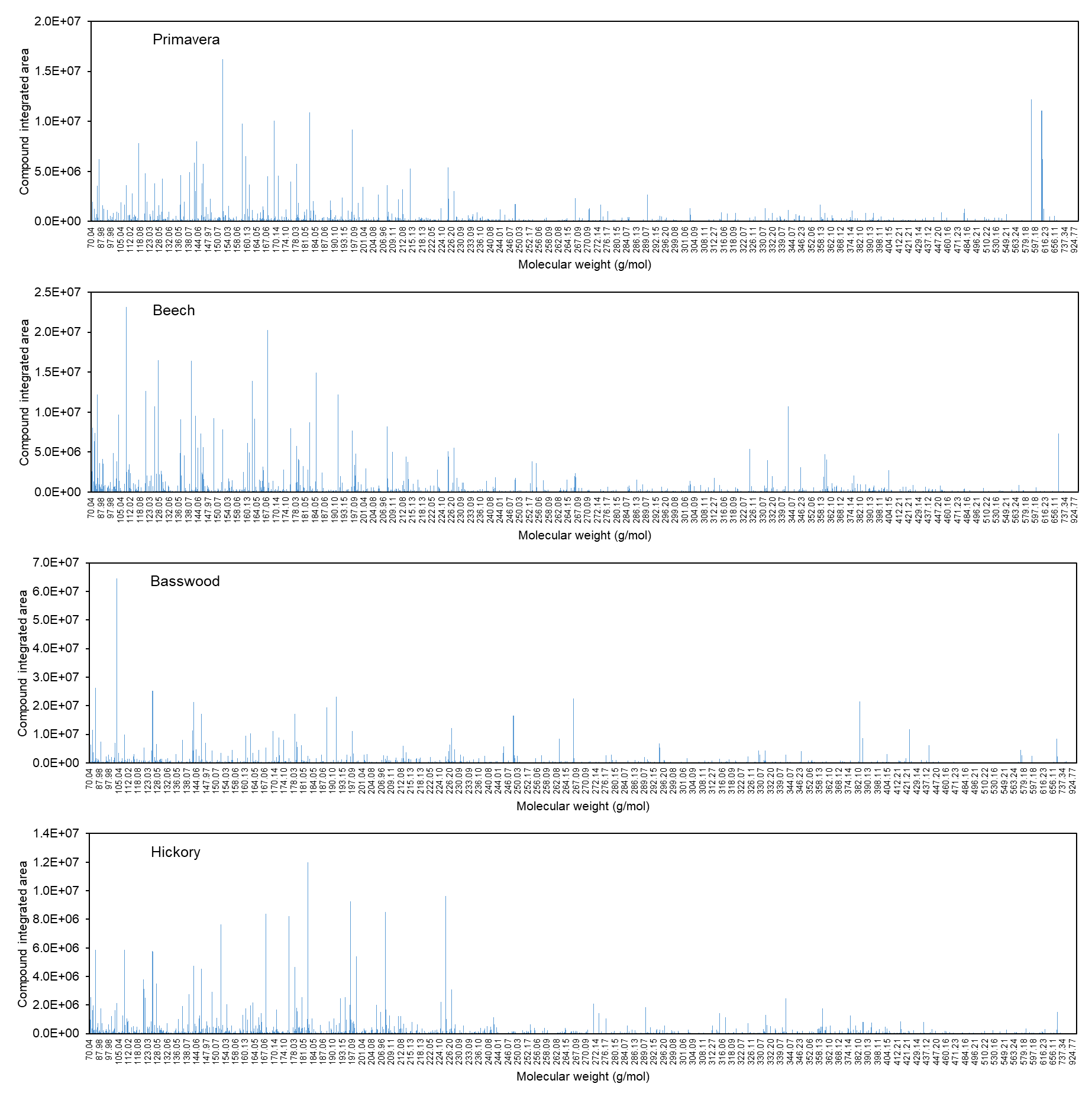


**Figure S3 (continued). Chemical compounds (identified by molecular weight and quantified by compound integrated area) in shavings from 30 different woods**


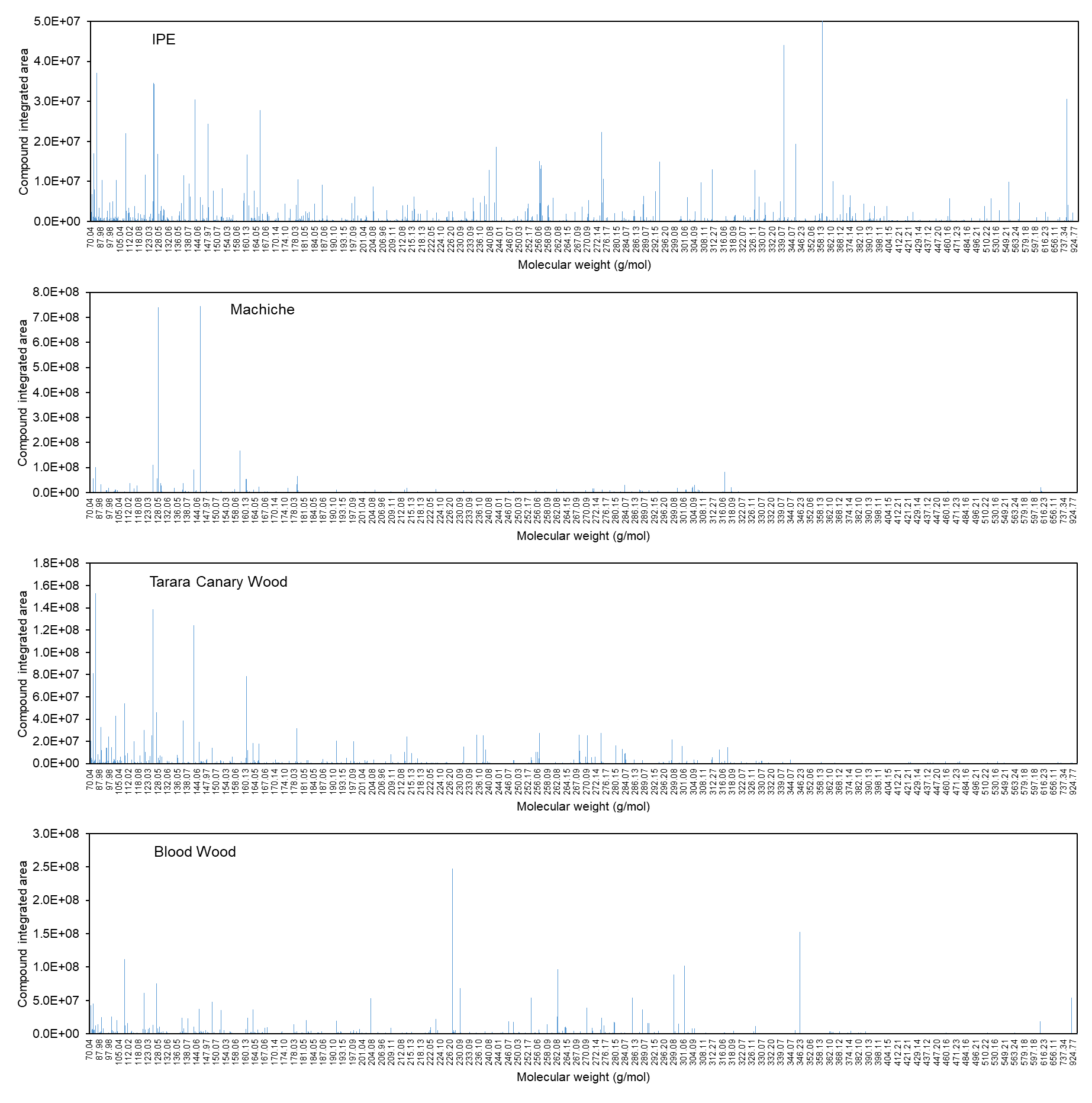


**Figure S3 (continued). Chemical compounds (identified by molecular weight and quantified by compound integrated area) in shavings from 30 different woods**


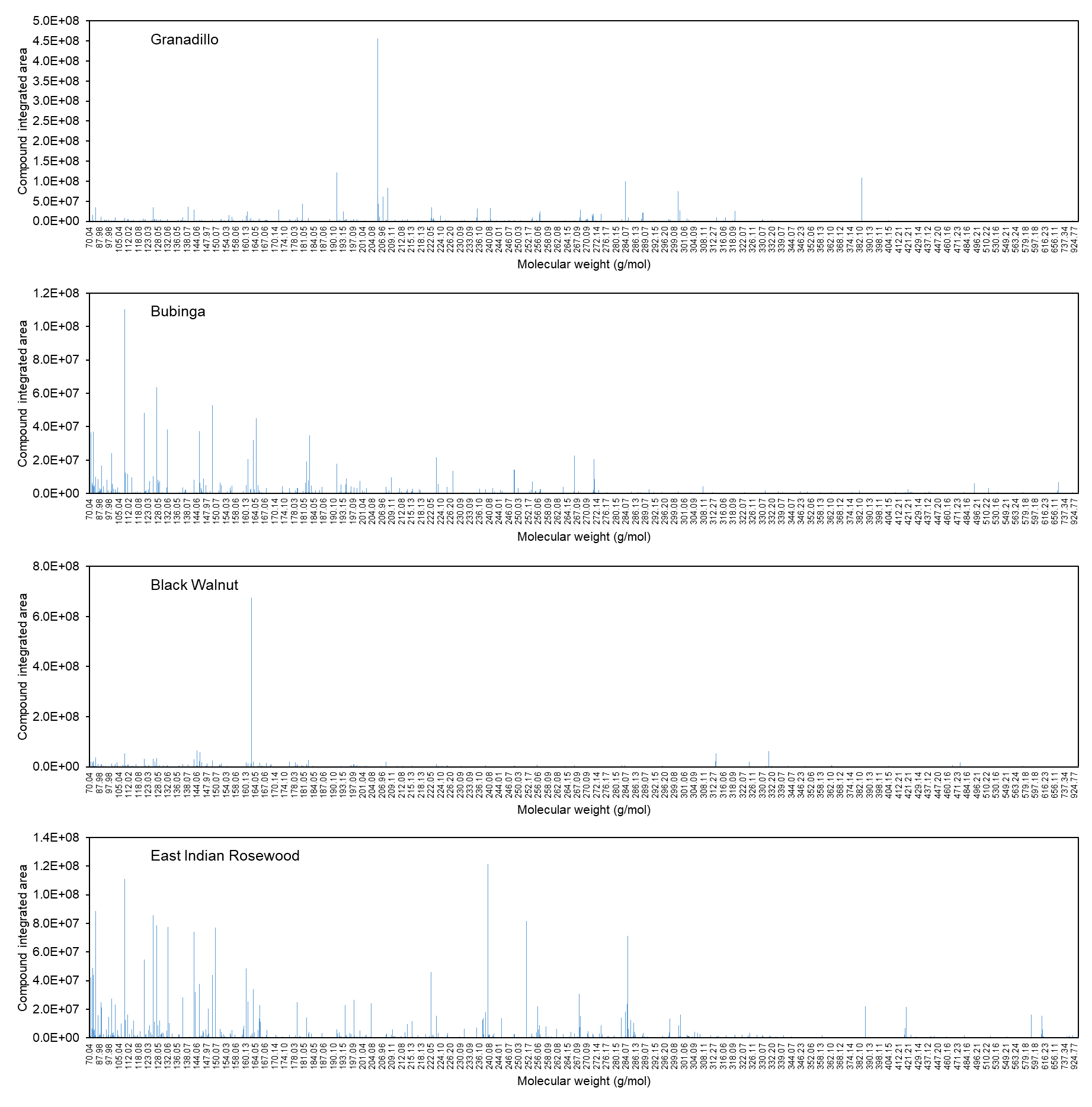


**Figure S3 (continued). Chemical compounds (identified by molecular weight and quantified by compound integrated area) in shavings from 30 different woods**


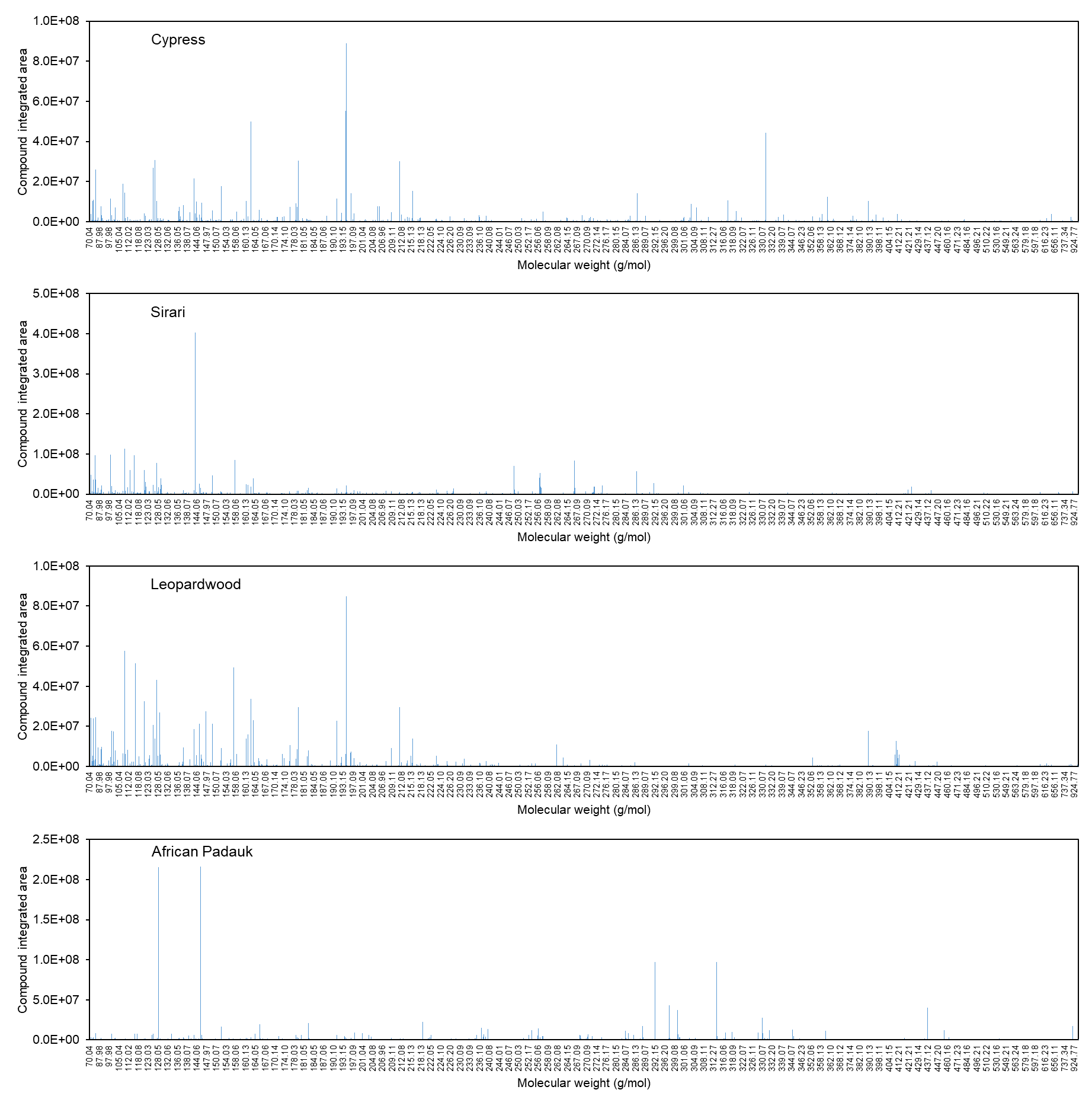


**Figure S3 (continued). Chemical compounds (identified by molecular weight and quantified by compound integrated area) in shavings from 30 different woods**


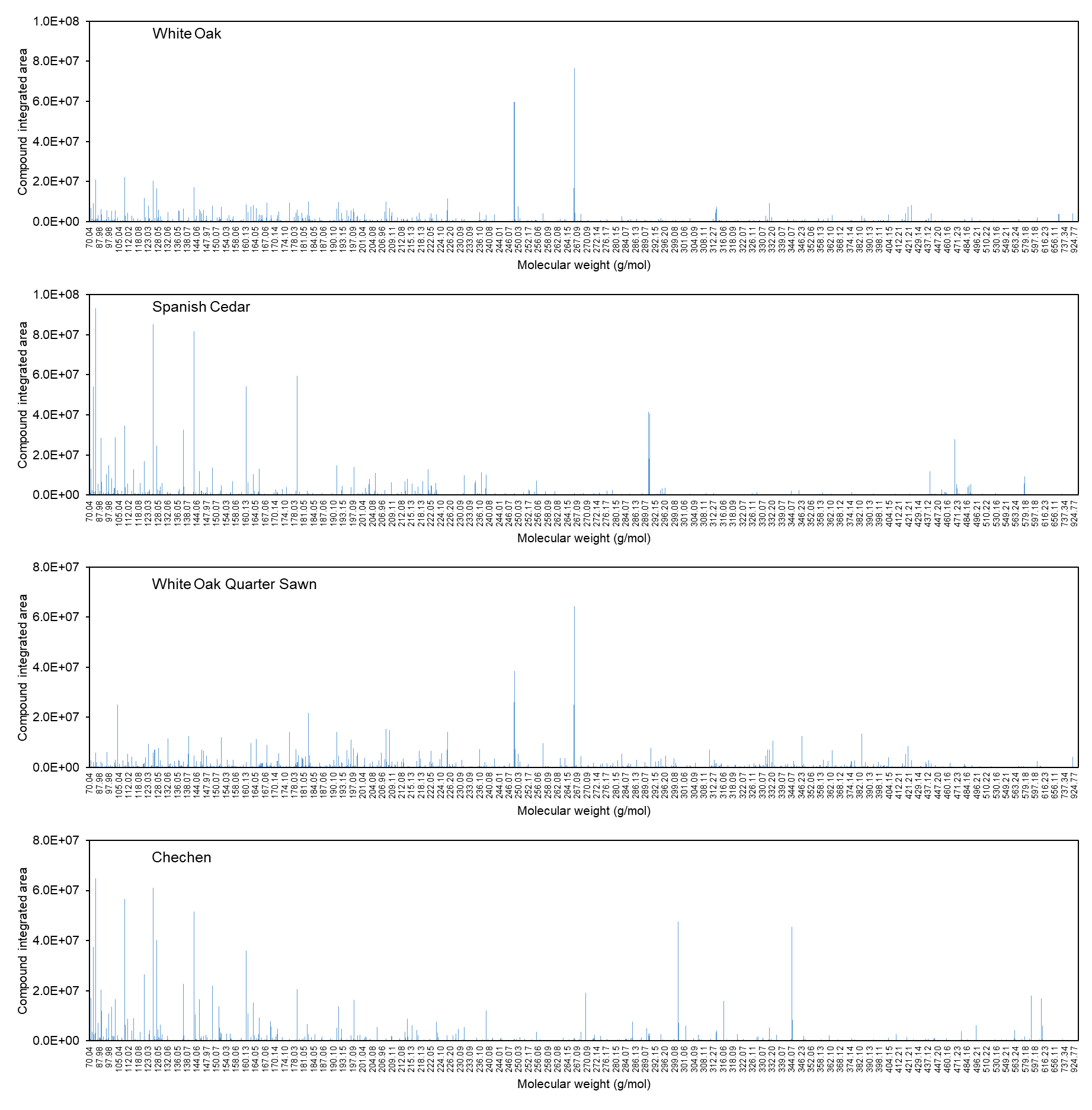


**Figure S3 (continued). Chemical compounds (identified by molecular weight and quantified by compound integrated area) in shavings from 30 different woods**


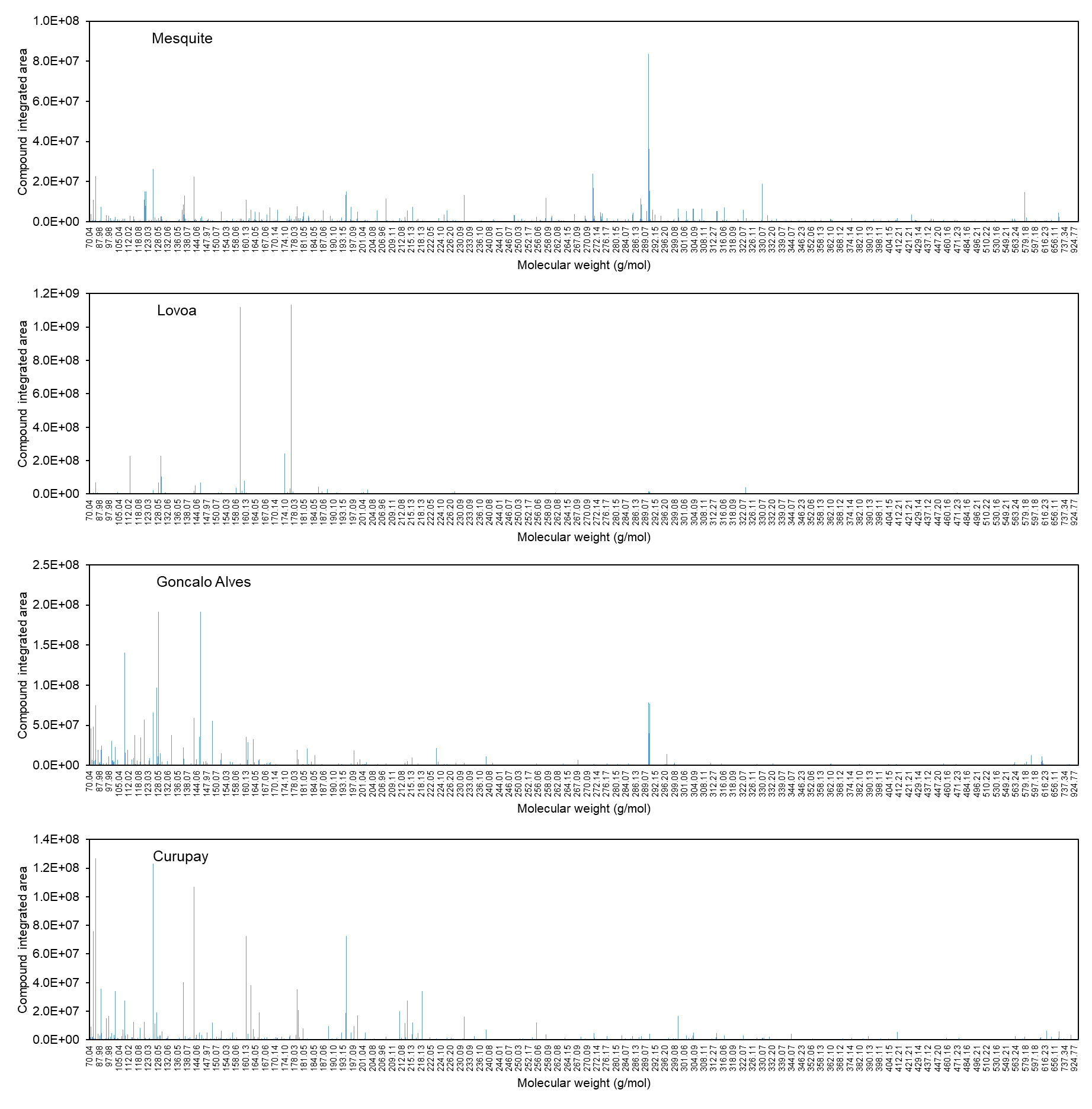


**Figure S3 (continued). Chemical compounds (identified by molecular weight and quantified by compound integrated area) in shavings from 30 different woods**


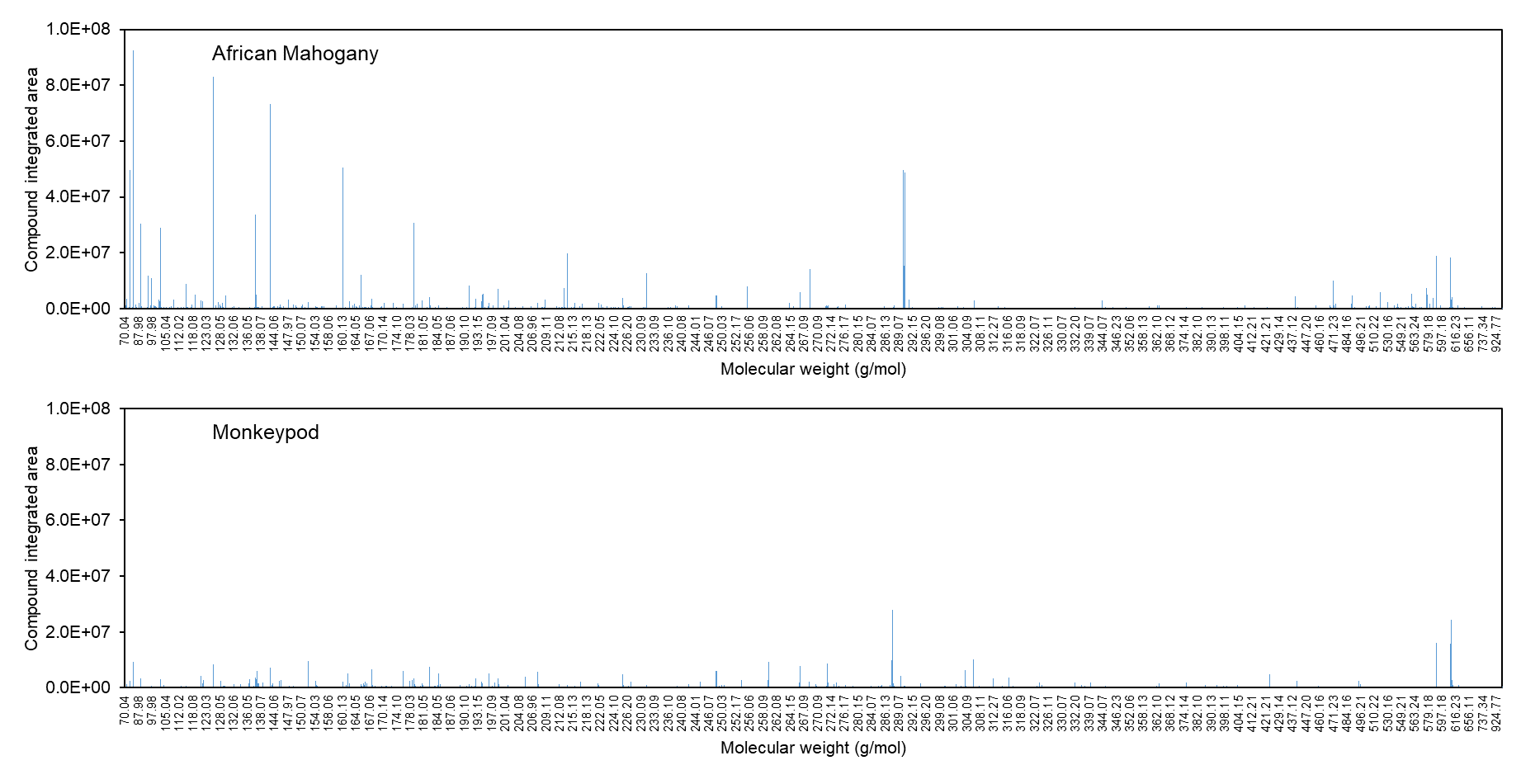


**Figure S3 (continued). Chemical compounds (identified by molecular weight and quantified by compound integrated area) in shavings from 30 different woods**

**
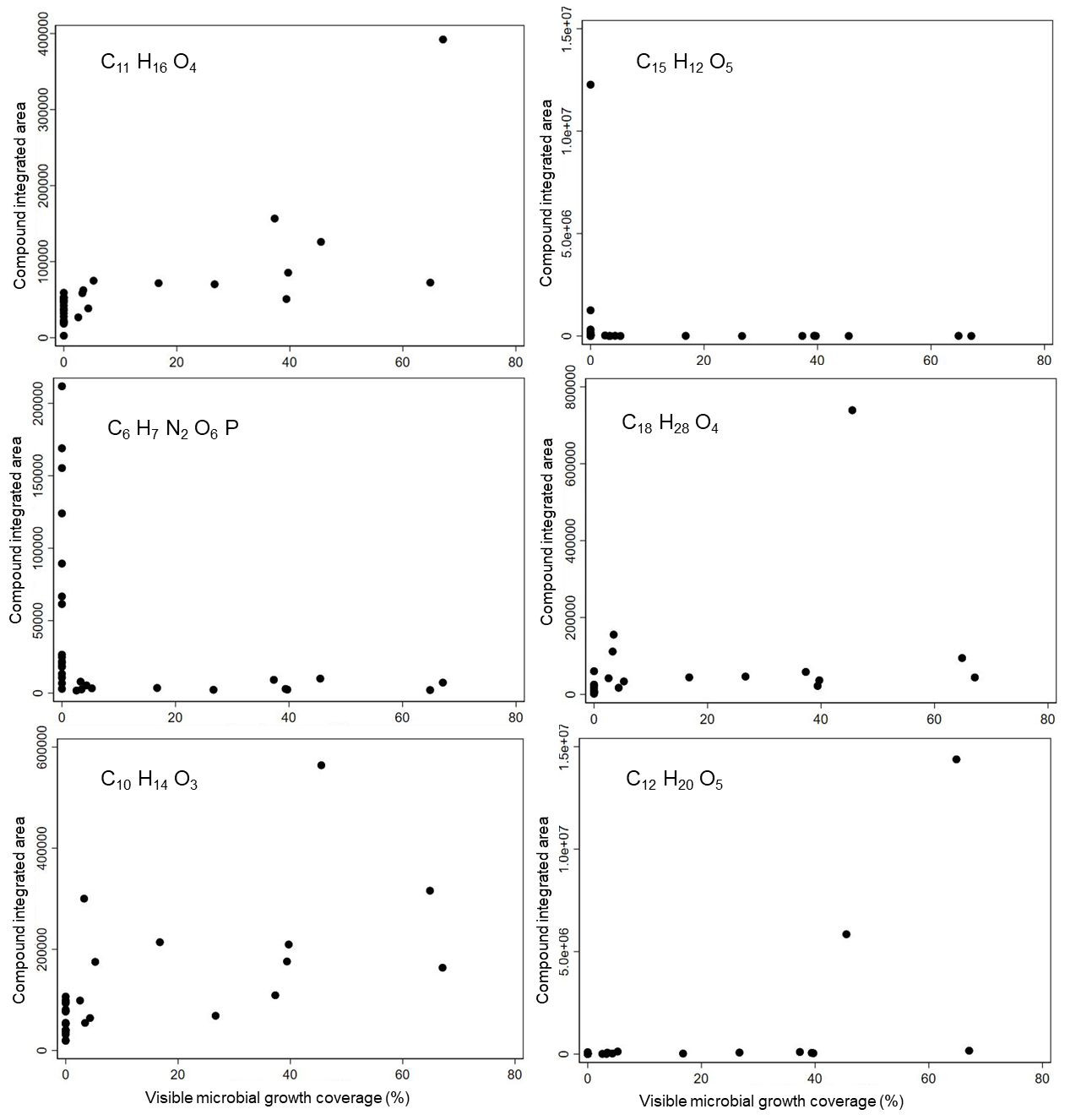
**

**Figure S4. Correlations between visible microbial growth coverage and the 35 chemical compounds with the strongest Spearman rank correlation coefficients with visible growth**

**
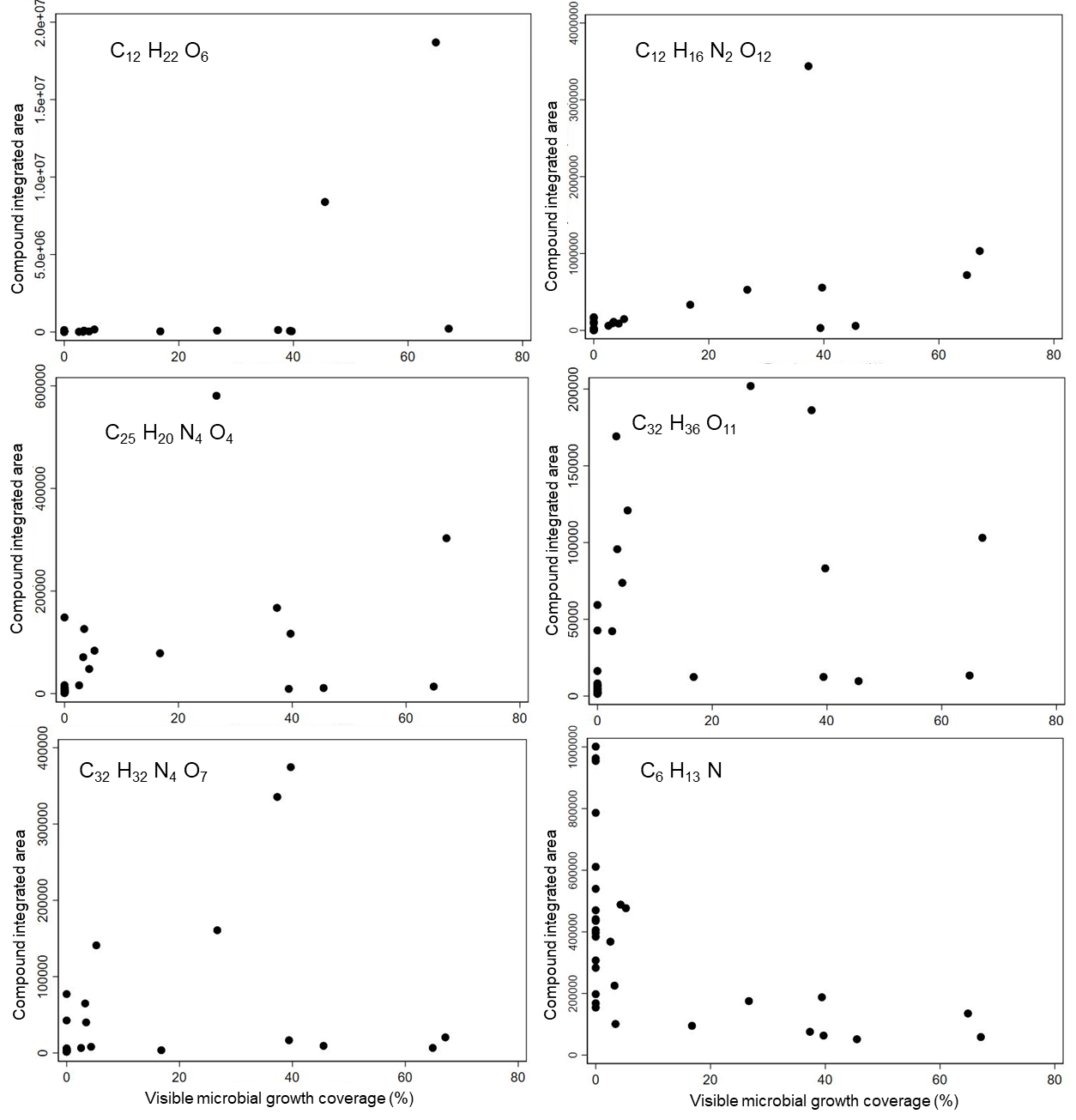
**

**Figure S4 (continued). Correlations between visible microbial growth coverage and the 35 chemical compounds with the strongest Spearman rank correlation coefficients with visible growth**

**
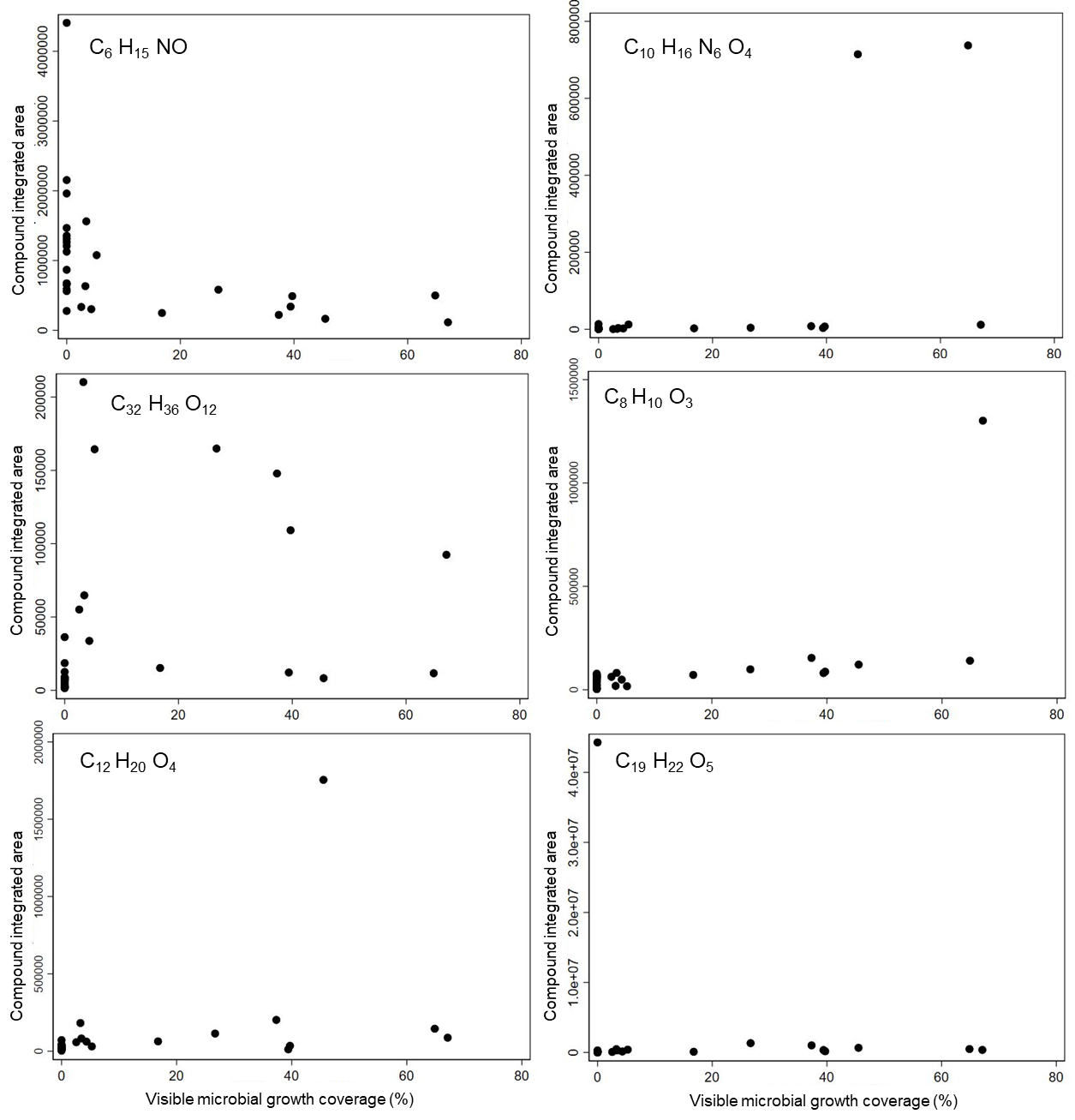
**

**Figure S4 (continued). Correlations between visible microbial growth coverage and the 35 chemical compounds with the strongest Spearman rank correlation coefficients with visible growth**


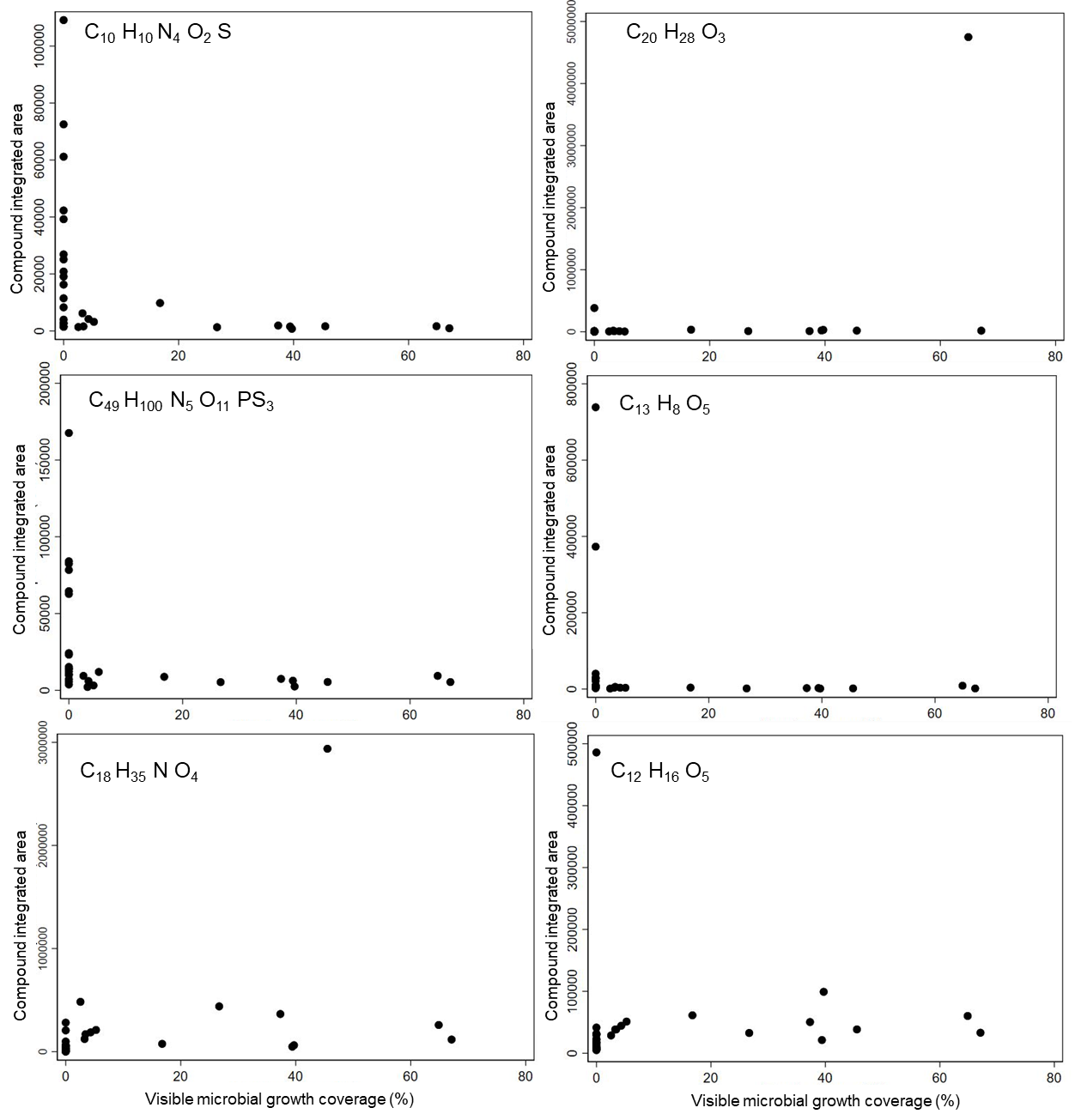


**Figure S4 (continued). Correlations between visible microbial growth coverage and the 35 chemical compounds with the strongest Spearman rank correlation coefficients with visible growth**

**
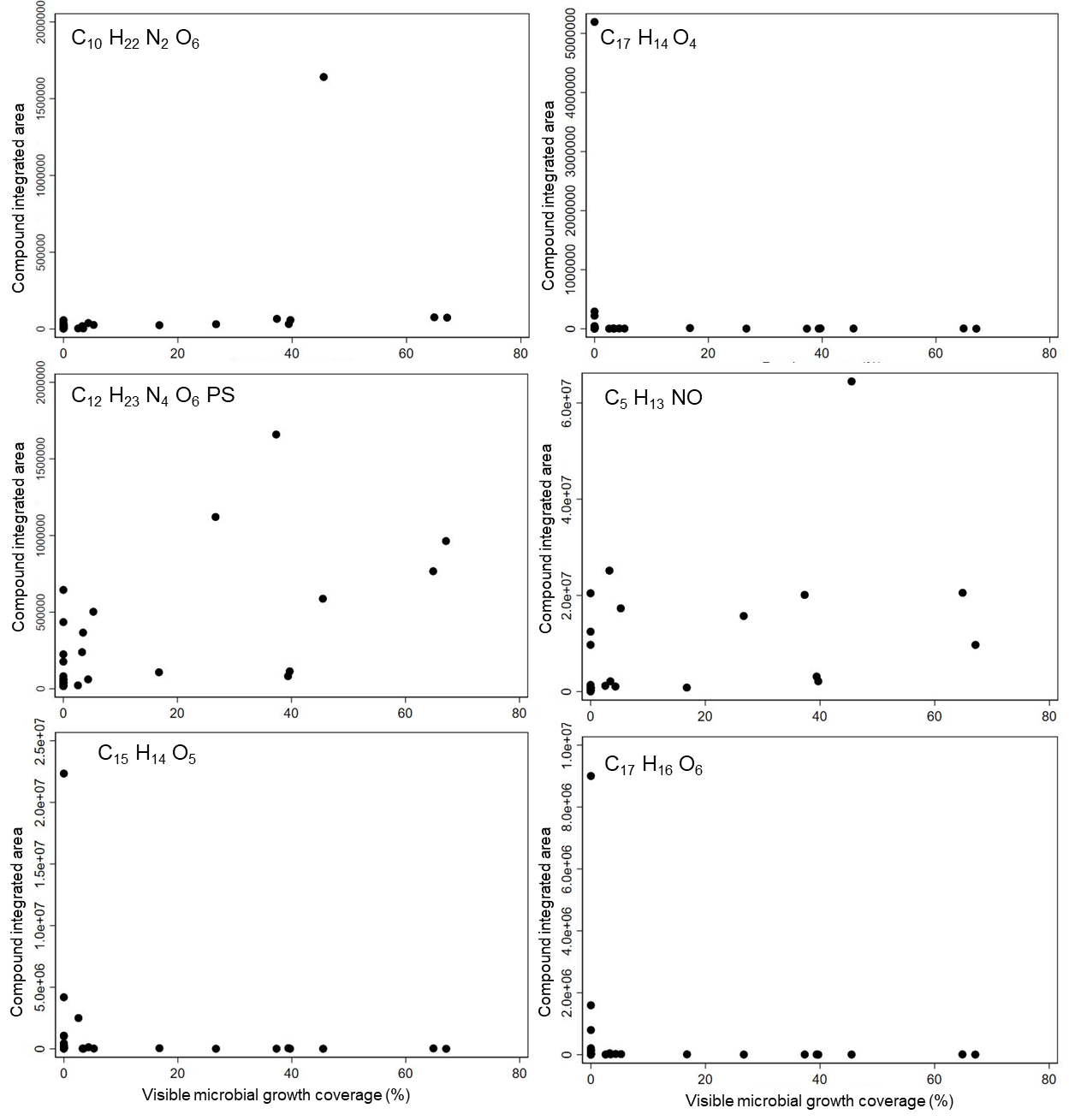
**

**Figure S4 (continued). Correlations between visible microbial growth coverage and the 35 chemical compounds with the strongest Spearman rank correlation coefficients with visible growth**


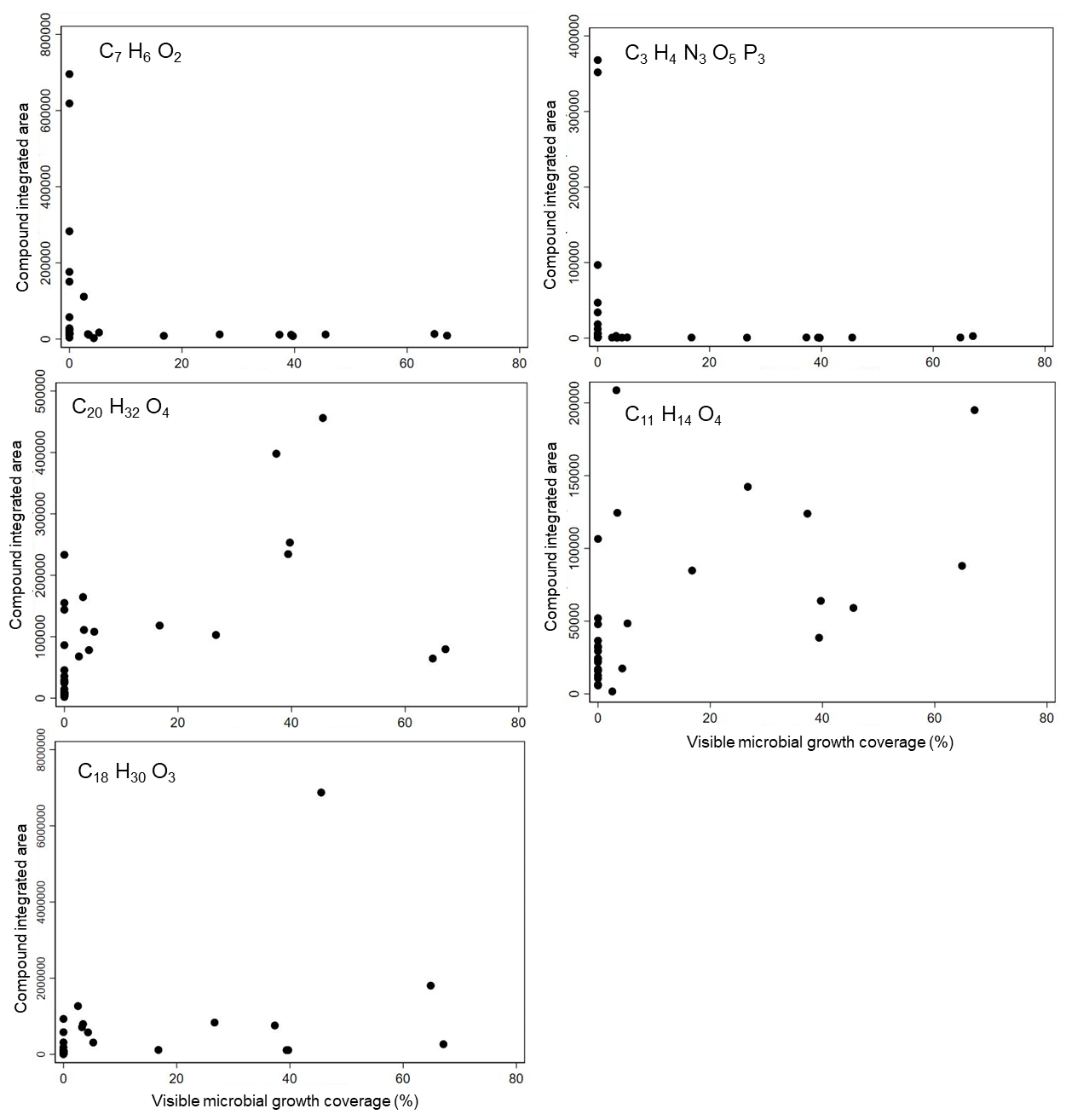


**Figure S4 (continued). Correlations between visible microbial growth coverage and the 35 chemical compounds with the strongest Spearman rank correlation coefficients with visible growth**

**
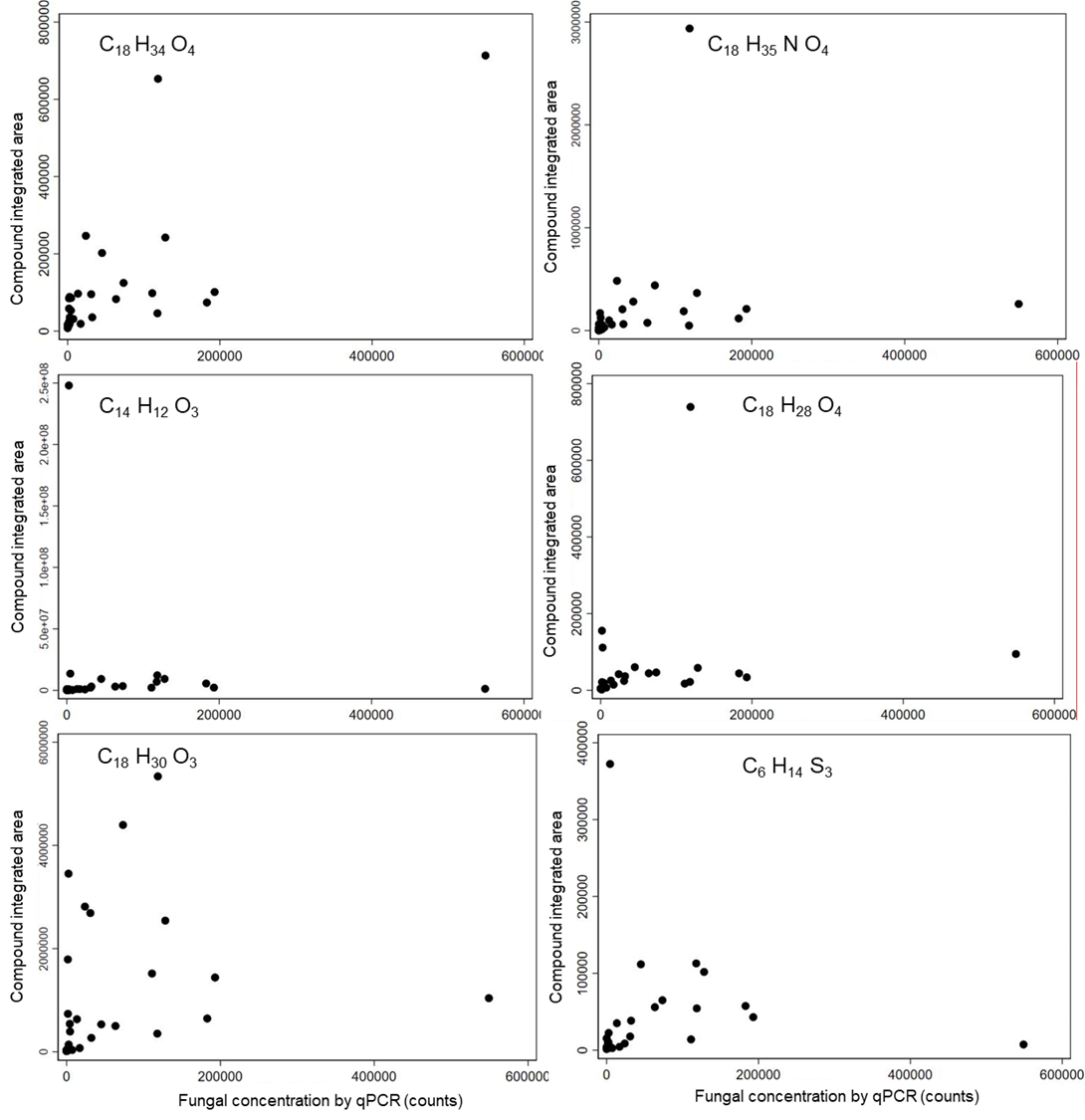
**

**Figure S5. Correlations between fungal qPCR concentrations and the 25 chemical compounds with the strongest Spearman rank correlation coefficients with fungal qPCR concentrations**

**
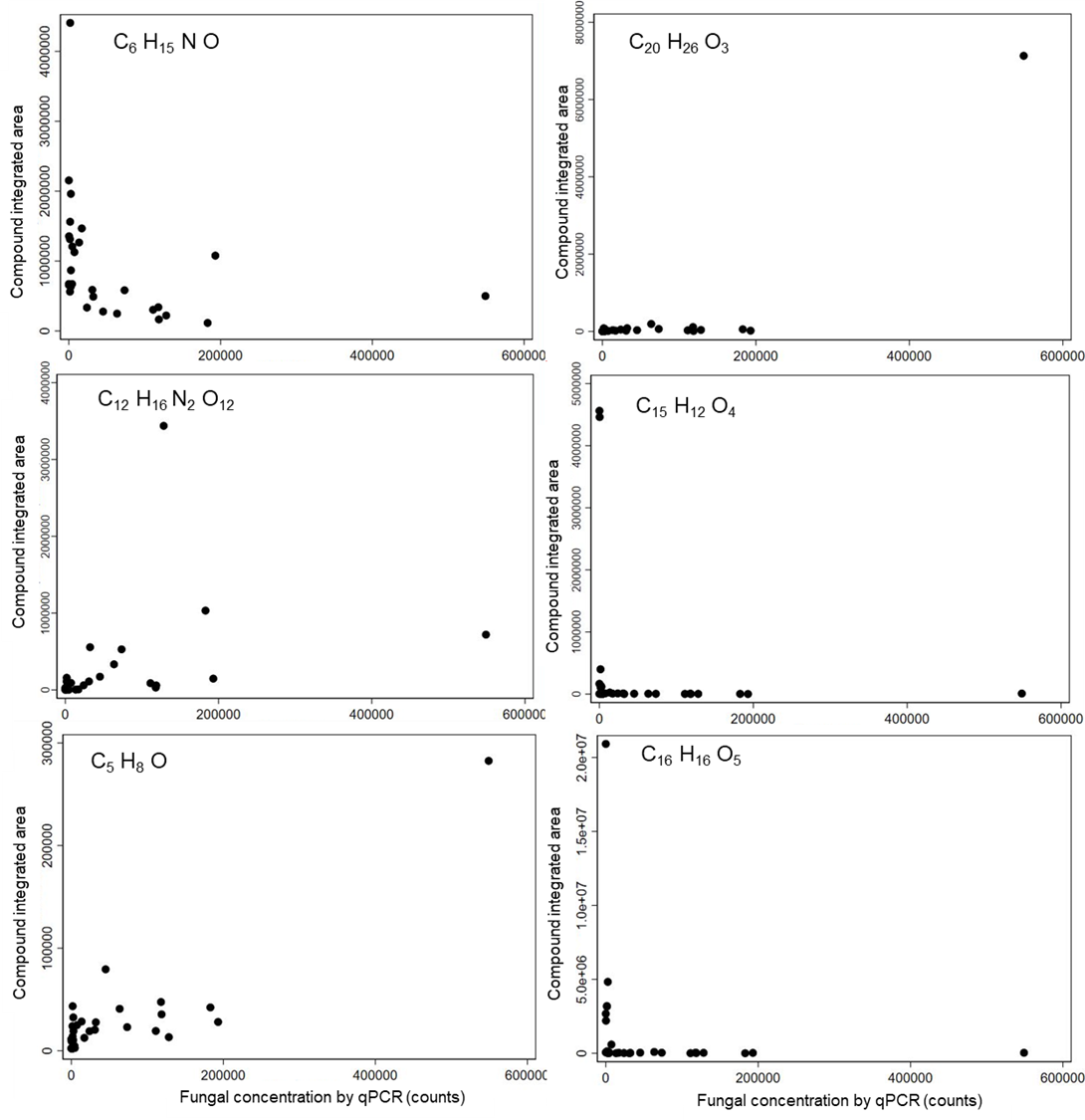
**

**Figure S5 (continued). Correlations between fungal qPCR concentrations and the 25 chemical compounds with the strongest Spearman rank correlation coefficients with fungal qPCR concentrations**

**
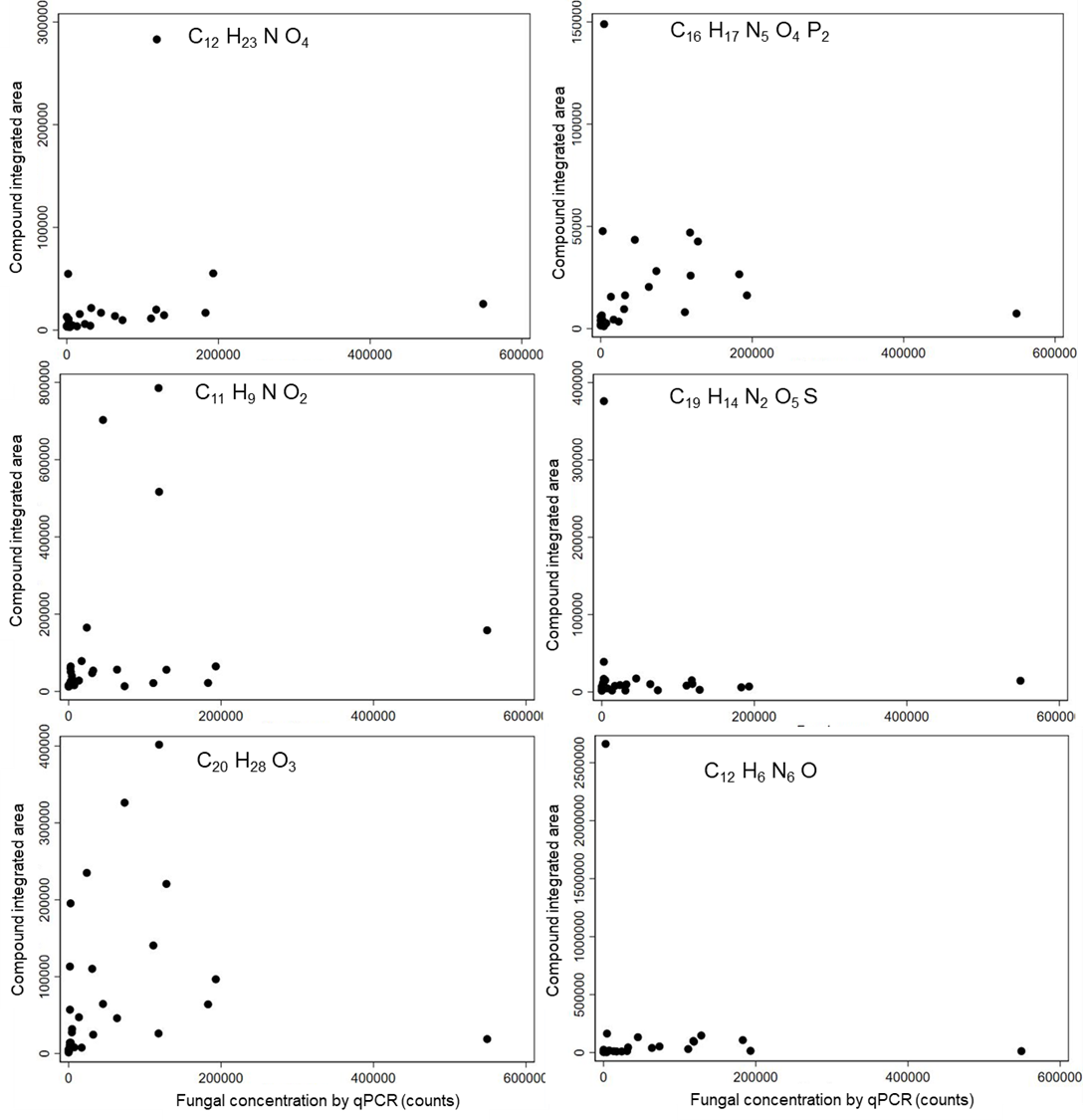
**

**Figure S5 (continued). Correlations between fungal qPCR concentrations and the 25 chemical compounds with the strongest Spearman rank correlation coefficients with fungal qPCR concentrations
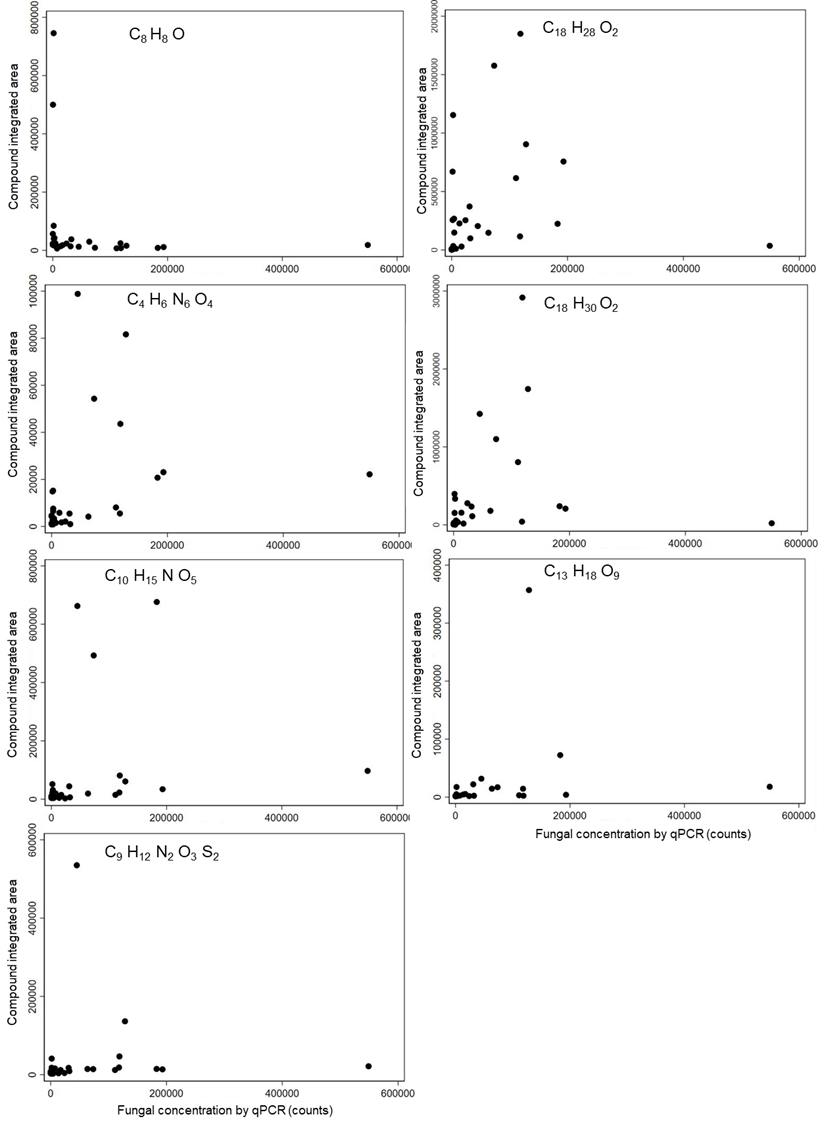
Figure S5 (continued). Correlations between fungal qPCR concentrations and the 25 chemical compounds with the strongest Spearman rank correlation coefficients with fungal qPCR concentrations**
